# Trace Element Concentrations Associated with Mid-Paleozoic Microfossils as Biosignatures to Aid in the Search for Life †

**DOI:** 10.3390/life11020142

**Published:** 2021-02-13

**Authors:** Andrew Gangidine, Malcolm R. Walter, Jeff R. Havig, Clive Jones, Daniel M. Sturmer, Andrew D. Czaja

**Affiliations:** 1 Department of Geology, University of Cincinnati, Cincinnati, OH 45221, USA; sturmedm@ucmail.uc.edu (D.M.S.); andrew.czaja@uc.edu (A.D.C.); 2 United States Naval Research Laboratory, Washington, DC 20375, USA; 3 School of Biological, Earth & Environmental Sciences, University of New South Wales, Kensington, NSW 2033, Australia; profmalcolmwalter@gmail.com; 4 Department of Earth and Environmental Sciences, University of Minnesota, Minneapolis, MN 55455, USA; jhavig@umn.edu; 5 Department of Earth and Planetary Sciences, Washington University in St. Louis, Saint Louis, MO 63105, USA; clivejones@wustl.edu

**Keywords:** biosignatures, biogeochemistry, trace elements, microfossils, hot springs

## Abstract

Identifying microbial fossils in the rock record is a difficult task because they are often simple in morphology and can be mimicked by non-biological structures. Biosignatures are essential for identifying putative fossils as being definitively biological in origin, but are often lacking due to geologic effects which can obscure or erase such signs. As such, there is a need for robust biosignature identification techniques. Here we show new evidence for the application of trace elements as biosignatures in microfossils. We found elevated concentrations of magnesium, aluminum, manganese, iron, and strontium colocalized with carbon and sulfur in microfossils from Drummond Basin, a mid-Paleozoic hot spring deposit in Australia. Our results also suggest that trace element sequestrations from modern hot spring deposits persist through substantial host rock alteration. Because some of the oldest fossils on Earth are found in hot spring deposits and ancient hot spring deposits are also thought to occur on Mars, this biosignature technique may be utilized as a valuable tool to aid in the search for extraterrestrial life.

## 1. Introduction

The search for ancient life has now expanded beyond Earth, notably with NASA’s Mars 2020 mission. The *Perseverance* rover has a primary mission objective of searching for evidence of past life and collecting samples which will be returned to Earth for analyses by a follow-up mission [1,2]. Verifiably determining the existence of life outside of Earth would be a paramount moment in scientific history with profound implications regarding the commonality of life in the Universe. Biosignatures (morphological, chemical, and mineralogical signs of life) are crucial for this task and are strongest when applied as a cascade of evidence, where multiple signatures connected to geochemical and geological context consistently support a biological interpretation. Even on Earth, many putative ancient fossils are controversial, highlighting the need for additional biosignature detection techniques [3,4,5,6,7,8,9]. As such, the scientific community must be prepared to adequately address the unprecedented burden of proof associated with the analyses of returned samples from Mars and/or other Solar System bodies.

Because not all environments preserve evidence of life equally and crucial biosignatures can be lost over time [10], there is a need to develop geologically robust biosignatures that have application in environments that are both likely to be encountered by the *Perseverance* rover (and future rovers/manned missions on Mars) and also yield a high preservation potential [11,12]. Jezero crater is thought to potentially host remains of impact-induced hydrothermal activity, where deep fractures could have given rise to terrestrial hot springs by channeling sub-surface magma to heat groundwater [13,14]. On Earth, hydrothermal spring deposits have been highlighted as environments having a high preservation potential with relevance to Mars [11,15,16,17]. These deposits form as hot spring water discharges and cools at the Earth’s surface, precipitating sinter formed primarily of amorphous silica (opal-A) [18]. The diagenetic alteration sequence of opal-A ends with quartz, which comprises many of the host rocks of the earliest putative fossils known on Earth [19,20]. Furthermore, terrestrial hydrothermal systems have been hypothesized as possible sites for the origin of life on Earth [21]. Combined with the tendency of such depositional environments to preserve evidence of life with high fidelity, hot springs are of prime astrobiological interest.

**Figure 1:**
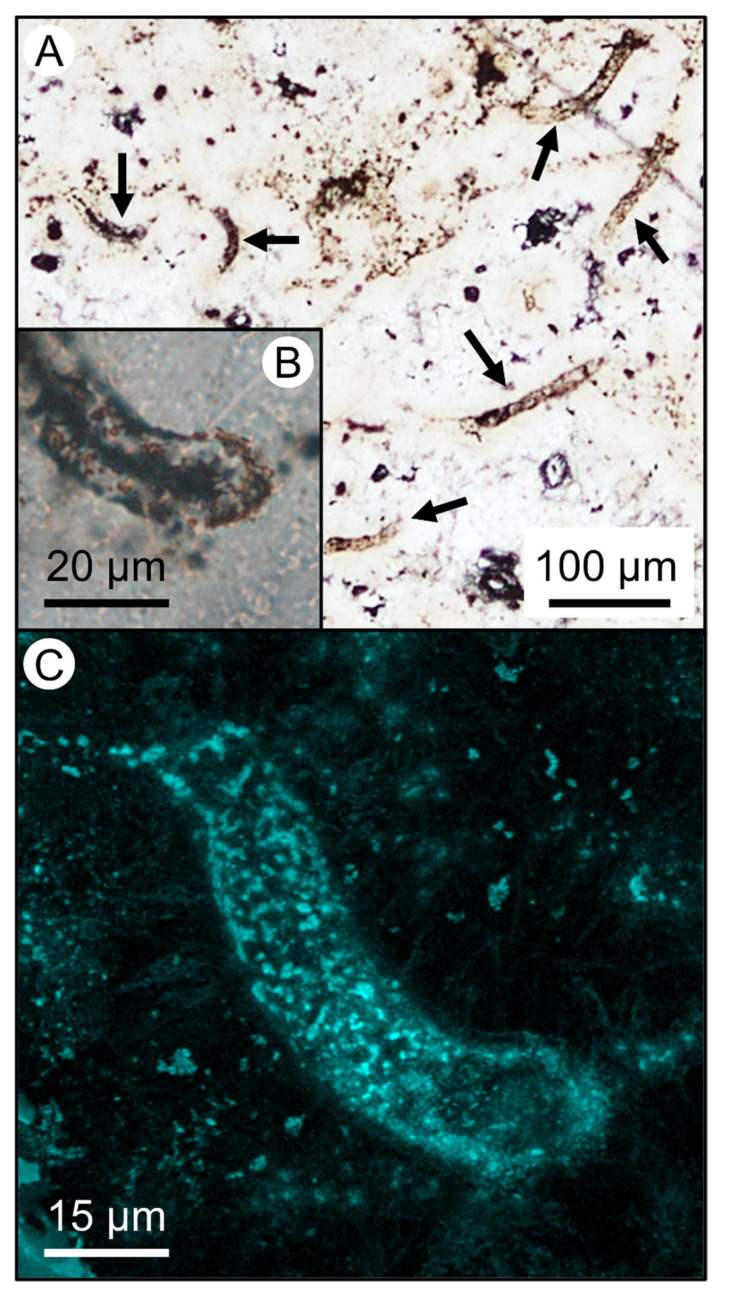
Microfossils of the mid-Paleozoic Drummond Basin, Queensland, Australia. (**A**) Plane-polarized light photomicrograph of several well-preserved filamentous microfossils, indicated by arrows. (**B**) Detailed view of a microfossil from panel A (indicated by the left-most arrow in panel A). (**C**) Confocal laser scanning microscope image of the microfossil from panel B, highlighting the organic cell walls via fluorescence.

The Late Devonian to Early Carboniferous sinters of the Drummond Basin in Queensland, Australia, host several mid-Paleozoic sequences and crop out over a ~25,000 km^2^ area [22] (Supplementary Materials). This site contains a range of microbial textures preserved in silica deposited from ancient subaerial hot springs, which have been previously reported and described [23,24]. The silica would have originally been deposited as amorphous opal-A and has since been diagenetically altered to microcrystalline chert, both polymorphs of SiO_2_. Several sites within the basin display putative cyanobacterial stromatolites and microfossils interpreted as cyanobacterial sheaths preserved in silica sinter deposited by terrestrial hot springs (as described in detail by prior studies) [23,24,25] (Figure 1), which serve as analogue environments for both ancient Earth and Mars. Microfossils in such hydrothermal environments often become entombed in a silica matrix by silica precipitating from hot spring waters, which can provide remarkable sample preservation throughout diagenesis (opal-A to quartz) [17]. The Drummond Basin has been previously described as a close paleoenvironmental match to modern sinters found in Yellowstone National Park, being of similar geologic setting and possessing homologous microfacies to those found in the thermal springs of Yellowstone despite a substantial difference in age and diagenetic state [23,24]. 

Sixteen microfossils from the Wobegong North sinters, located within the Conway Hydrothermal System in Drummond Basin, displaying thin-bedded palisade fabrics (a common texture of silica sinter formed by populations of filamentous microorganisms [26]), were chosen for analyses [24]. Microfossils were located and imaged via transmitted light microscopy (Supplementary Materials) and were analyzed via confocal laser scanning microscopy to confirm features commonly associated with microbial remains (e.g., fluorescent cell walls, hollow structure—see Figure 1). These microfossils were analyzed for trace element distributions of Mg, Al, Cr, Mn, Fe, Ga, Sr, As, Sn, and Sb colocalized with C and S (elements which comprise or are commonly associated with microbial remains) using a Cameca IMS 7f-GEO secondary ion mass spectrometer (SIMS), which allowed for the detection of trace elements even at low abundances, and the generation of micron-scale elemental maps. These elements were chosen for study based on a variety of factors including metabolic relevance, known presence in modern hydrothermal spring waters [27] and in Drummond host rock samples (Table 1), and known sequestrations in analyses of microbial remains in modern to sub-recent hot spring deposits in Yellowstone National Park [28]. Prior work has shown that various elements, including many of which are generally abundant and naturally occurring in hydrothermal systems through inorganic means (and likely not metabolically relevant), are still preferentially enriched in microbial communities [28,29]. For example, Al naturally occurs in many hot spring systems and is highly immobile in most surface environments [30]. It can, however, become mobile in acidic hot spring fluids, making it difficult to deduce whether any association of Al with microfossil remains is due to a natural sorption to organic matter or a more complex process whereupon dissolved Al may be incorporated inside microorganisms through other means. Springs may also be naturally enriched in certain elements while active (e.g., Fe/Mn-rich springs), and other elements may be concentrated during late diagenesis/alteration.

The analyses performed in this study are primarily intended to test whether any elemental sequestration trends observed in less diagenetically mature samples from Yellowstone hot spring deposits also occur in the older and more heavily altered Drummond deposits despite substantial host rock alteration [28,29]. More generally, this will add further data to the mounting evidence that suggests the potential biosignature application of trace elements for both ancient Earth and Mars [31,32,33,34,35,36,37], and the utility and merit of analyses focusing on trace elements for martian in situ and sample return analyses.

## 2. Materials and Methods 

Samples were cut and polished using a Hillquist thin section machine, further polished using a ~70 µm grit polishing wheel and were then mounted on 1-inch round glass slides using Hillquist thin section epoxy. Samples were then cut to ~100 µm thicknesses for optical photomicrography. Samples were polished to a ~2 µm (peak to valley) smoothness, and then imaged via transmitted light microscopy using an Olympus BX-41 microscope (UPlanFl 10× and 20×, numerical apertures of 0.30 and 0.50 respectively) and an Olympus SC30 digital camera. Raman analyses were performed using a Horiba T64000 Raman microscope at the University of Cincinnati.

SIMS analyses were carried out using a Cameca IMS 7f-GEO system at Washington University in St. Louis using previously described methods [28]. Major and trace elements were measured as negative ions (produced by Cs^+^ primary ion bombardment) and positive ions (produced by O^−^ primary ion bombardment), respectively, to provide optimum sensitivity. Silicon was also included with each positive secondary ion image of trace elements to facilitate positive and negative ion image coregistration. The primary beam current and net impact energy was 25 pA (20 keV) and 140 pA (23 keV) for Cs^+^ and O^−^, respectively, and the beam diameter was 1–2 µm in each case. Mg, Al, Cr, Mn, Fe, Ga, and Sr were measured using O^−^, and C, CN^−^, P, S, As, and Sb were measured using Cs^+^. With a mass resolving power of 3000, most interferences could be confidently characterized, with a few exceptions noted in the Supplementary Materials. It is notable that SIMS images may visually vary based on the specific element of interest and beam species used in any given analysis, as well as other factors such as elemental uniformity within a sample, and should not be viewed as quantitative on their own.

Quantification of the trace element data was achieved by analyzing National Institute of Standards and Technology (NIST) standard reference materials 610 and 617 (glass reference materials containing known amounts of trace elements) and synthetic glass standards WU-A and WU-B [38]. For the elements analyzed, the 95% confidence level in the documented relative concentration value of the reference materials did not exceed 10% (e.g., a concentration of 10 mg/kg would be within ±1 mg/kg). The NIST reference materials were analyzed utilizing identical analytical parameters to those used for the unknowns. These data were then used to calculate a sensitivity factor for each element, which varies due to the electron affinity of each element [39], and was then applied to the raw sample data to calculate the concentration of each element (see Supplementary Materials for data). Prior analyses of float zone silicon, a high-purity form of silicon which provides an analogous moving region of stoichiometry to silica via the implantation of O^−^ by the primary beam, showed that instrumental detection limits were sufficient for the proposed analyses [28]. X-ray fluorescence (XRF) analyses of Drummond Basin chert were performed on fused beads by a Rigaku Supermini 200 wavelength dispersive XRF instrument. Confocal laser scanning microscopy was performed with an Olympus Fluoview 1200, a 60× oil immersion lens (numerical aperture [NA] = 1.42), and an excitation laser line of 488 nm.

## 3. Results and Discussion

**Figure 2:**
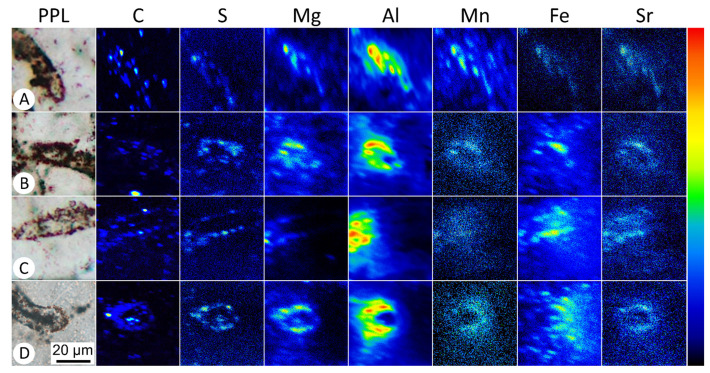
SIMS mapping of select Drummond microfossils. The first column (**A**–**D**) shows plane-polarized light (PPL) images of microfossils targeted for SIMS analyses, with subsequent rows showing the elemental map for the noted element. See Supplementary Materials for complete SIMS image data and quantified concentrations of each sample. The color bar on the right shows relative values from low (black) to high (red). Scale bar in panel D applies to all panels. Note that increased elemental concentrations relative to background are indicated only where the fossil intersects the plane of the top of the thin section, as SIMS is a surface-analysis technique.

**Figure 3:**
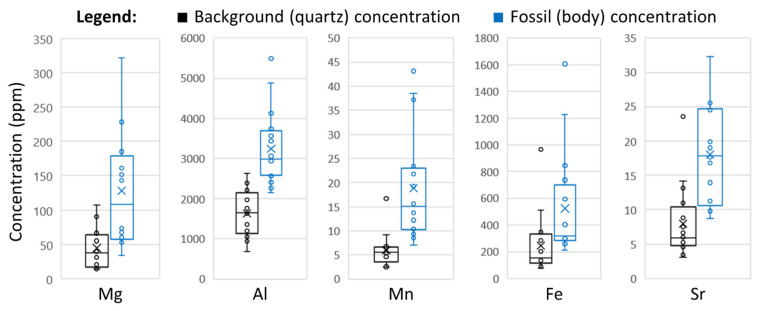
Trace element concentrations of the 16 individual Drummond microfossils (blue plots) compared to the background trace element concentrations in the same sample (black plots). The background is defined as the silica matrix of the sample which does not overlap any microfossil or other apparent organic structure. Y-axis shows elemental concentration in parts-per-million. See Supplementary Materials for full dataset. Each individual background/quartz concentration obtained was lower than the corresponding fossil concentration (see Supplementary Materials). Plots denote the range of data (including outliers), mean data (denoted by ×), and boxes showing the first quartile, median line, and third quartile data.

Among the fossils analyzed, several trace elements were found in consistent distribution patterns. Mg, Al, Mn, Fe, and Sr showed consistent elevated concentrations within microfossil bodies (Figure 2), with each microfossil exhibiting a higher concentration of each respective element than the background concentration in the surrounding quartz matrix (Figure 3; Supplementary Materials). Raman analysis of microfossils showed only the presence of kerogen and quartz in the acquired spectra, suggesting a substantial mineral-control for the observed elemental distributions is not likely (Supplementary Materials). Aluminum showed consistently high concentrations colocalized with filament bodies, with calculated concentrations ranging from 2 to 6% (average = 3.2%). XRF analyses of the host rock verified that Al was indeed naturally abundant in the host rock, at nearly 1.7% (Table 1, Supplementary Materials). Within the fossils, magnesium was found in concentrations ranging from ~30 to 350 ppm (average = 128 ppm), manganese in lower concentrations ranging from ~7 to 50 ppm (average = 19 ppm), iron in concentrations ranging from ~200 to 1600 ppm (average = 524 ppm), and strontium in concentrations ranging from ~8 to 35 ppm (average = 18 ppm).

Visually, apparent sequestration patterns for Cr in microfossil bodies can be noted in some samples (Supplementary Materials), but similarly to prior studies, the lack of adequate counts obtained do not allow for these data to be quantified [28]. Likewise, no general sequestration trends could be determined for Ga, Sn, or Sb. Gallium has previously been found to be associated with microbial filaments in modern hydrothermal systems, although the presence of gallium was only observed surrounding the microbial filament as opposed to being colocalized with the body of the microorganism. Tin was chosen to test if an element present in bulk host rock analyses (mass percent = 0.0044%) that is not biologically relevant and not generally found in hot spring water may still be associated with the microbial fossils, thus its absence is not surprising. Antimony was chosen as it is commonly present in modern hot spring water [27]. It has also been observed to be sequestered in some modern to sub-recent preserved microorganisms in Yellowstone (e.g., Supplementary Materials), but was one of the least robust trace elements studied, so its absence in the Drummond samples is also not surprising. 

It is important to note that microbial communities can associate with elements in many different direct and indirect ways. For example, some elements such as Fe and Mg are commonly utilized by enzymes and may be expected to be associated with microbial remains. Other elements (e.g., Al, Ga, Sr), which are not typically metabolically relevant, may indirectly interact with microorganisms through processes such as iron scavenging and subsequently be sequestered [40]. It is for this reason (with support from past research showing unexpected elemental sequestration trends associated with live microbial communities in modern terrestrial hot spring environments [28,29]), that a variety of elements were chosen for this study, despite some having more obvious biological relevance. It should also be noted that other research concerning trace element distributions associated with microorganisms in hot spring areas did not suggest preferential affiliation with microbial remains [41]; thus, factors leading to this discrepancy should be investigated.

Whereas the geologic setting of the Drummond sinters has been previously described [23,24], the composition of the host waters in this system is not well defined. As such, it is not clear whether the absence of any given element is due to poor preservation or whether these elements were not present/abundant in the original hydrothermal system or resulted from post-depositional alteration (which is thought to have occurred in many Drummond Basin deposits). Alternatively, a difference in metabolic activity may have led one group of organisms to associate with some elements but not others. Further work is required to fully tease apart the various possibilities leading to active or passive (e.g., biological versus abiological/mineralogical) elemental retention, and will be helpful to constrain possible biological controls on elemental sequestrations and the specific roles of various elements as potential biosignatures (e.g., [37]). It will also be helpful to further investigate the possible mechanisms (e.g., diagenesis, remobilization, etc.) responsible for the varying patterns of sequestration observed in some elements which show less well-defined fossil-to-background boundaries. 

Despite uncertainties in the initial trace elemental composition of the Drummond Basin hot spring waters, these results are consistent with trace element abundances found in association with preserved microorganisms in modern to sub-recent spring deposits in Yellowstone National Park, which contain microbial filaments with elevated trace element concentrations in and around their remains, relative to the surrounding silica matrix ([28]; Supplementary Materials). These results also add new elements of interest as potential biosignatures which were not previously analyzed in microbial body fossil remains. Whereas the exact metabolisms and modes of life of the Drummond microfossils are not known aside from apparent evidence of photosynthetic activity [24], these results highlight the broader implication that microbial fossils in hot spring systems may preserve unique, elevated concentrations of various elements that may have use as a biosignature, even when the fossil and host rock have been subjected to substantial diagenetic alteration. The Drummond microfossils are well suited for demonstrating the application of this biosignature, because the biological morphology and preserved organic material present means that the biogenicity of these microfossils would likely not be called into question.

Due to the repeated sequestration patterns in each microfossil analyzed, we propose Mg, Al, Mn, Fe, and Sr as having potential biosignature application for microbial life preserved in terrestrial hot spring deposits and being worthy of future analyses to better constrain their possible utility as biosignatures. As trace elemental sequestration trends have been shown to have the potential to persist in diagenetically mature samples with well-preserved microfossils, this biosignature could be applied to other hydrothermal chert samples with less well-preserved putative body and trace microfossils such as stromatolites, which have been the subject of investigation by other studies [37,42,43,44]. Obtaining similar trace element concentrations as those highlighted in this study may point to a possible biological origin of morphologically ambiguous textures. It is already known that hot spring microbial communities sequester distinct concentrations of various elements during life which differ from the sinter/host rock composition [29]. Each element likely has a unique sequestration potential and history that can be caused or affected by biological phenomenon such as specific microbial metabolisms and/or geologic/geochemical occurrences, both at the time of deposition and through subsequent alteration. Future studies on these topics will inform the application of specific elements to be utilized as biosignatures. Given sufficient understanding of sequestration trends in particular environments and by specific microbial communities during life and following preservation, it may be possible to obtain a cascade of evidence that convincingly argues for or against a biological interpretation of potential microbial fossils on Earth and from extraterrestrial samples.

Mapping trace element distributions with SIMS allows for high-resolution in situ elemental data to be obtained, and the ability to directly compare elemental retention and trends among samples of varying ages and geologic settings. The implications of robust, biologically associated and retained elemental sequestrations throughout time directly benefits the search for past life, as comparisons of elemental sequestrations in modern versus ancient samples could assist in ascribing metabolic/ancestral similarities. Any such insight gained as to the origin and early evolution of life on ancient Earth not only informs the understanding of our planet’s history, but also informs strategies for searching for life elsewhere in the Universe.

The search for evidence of life on Mars is already underway and bears the potential to inform questions regarding the commonality of life in the Universe and to facilitate a new era of planetary exploration. Should such evidence exist and be discovered, the confident interpretation of past life will require robust biosignatures that can persevere despite host rock alteration resulting from geologic processes and the passage of time. As such, we advocate the potential of trace elements as biosignatures as a continued subject of investigation and refinement for studies concerning Mars and ancient Earth, and suggest that this particular SIMS-based analytical method may be a valuable tool to be added to the suite of analyses of precious samples collected from Mars by the Mars 2020 mission and future sample return missions. 

## Figures and Tables

**Table 1:** Bulk Host-Rock X-ray Fluorescence.

Component	Mass %
SiO2	95.7379
Al_2_O_3_	1.6859
Fe_2_O_3_	0.2517
MgO	0.2249
Sr	0.012
MnO	0.0085

## Supplementary Materials

The following are available online at https://www.mdpi.com/2075-1729/11/2/142/s1, Figure S1. Geologic map of Drummond Basin, showing the location of the Wobegong North sinters within the Conway Hydrothermal System, where the samples from this study were collected (modified from Walter et al. 1996). Figure S2. SIMS analyses of a <10,000-year-old cyanobacterial microorganism (noted by red arrow) preserved in a hot spring deposit in Yellowstone National Park, showing similar elemental sequestration trends (notably Sr, Fe, Mg) as to what was observed in Drummond samples (see also Gangidine et al. 2020), but also displaying unique trends (e.g., Gallium). Figure S3. Visual graph illustrating the same data as Figure 3 in the main text, with each background value (left point) attached to the fossil concentration (right point) to show the increased concentration in each fossil analyzed. Figure S4 (1–16). The above photomicrographs show the 16 Drummond Basin microfossils analyzed in this study. The following pages show the SIMS images generated for each microfossil. Each panel shows a greyscale SIMS image for the noted element. Figure S5. XRF Heavy spectrum from Drummond Basin sample corresponding with Table 1 in the main text. Figure S6. XRF Total spectra from Drummond Basin sample corresponding with Table 1 in the main text. Figure S7. Drummond microfossil targeted by Raman analyses. Figure S8. Non-baseline subtracted Raman spectrum from Drummond microfossil body (pictured in Figure S7), showing strong fluorescence. Figure S9. Baseline subtracted Raman spectrum from Figure S8, showing quartz and kerogen. Smaller peaks are due to fluorescence. Table S1. SIMS interferences and comments for various masses. Table S2. Elemental data for 16 fossil samples and the surrounding mineral matrix (background) plotted in Figure 3 of the main text.

## References

[1] Farley K.A., Williford K.H. (2017). Seeking Signs of Life, and More: NASA’s Mars 2020 Mission. Eos.

[2] Mustard J.F., Adler M., Allwood A., Bass D.S., Beaty D.W., Bell J.F., Brinckerhoff W., Carr M., Des Marais D.J., Brake B. (2013). Report of the Mars 2020 Science Definition Team. Mars Explor. Program Anal. Group (Mepag).

[3] Muscente A.D., Czaja A.D., Tuggle J., Winkler C., Xiao S. (2018). Manganese Oxides Resembling Microbial Fabrics and Their Implications for Recognizing Inorganically Preserved Microfossils. Astrobiology.

[4] Wacey D., Noffke N., Saunders M., Guagliardo P., Pyle D.M. (2018). Volcanogenic Pseudo-Fossils from The ∼3.48 Ga Dresser Formation, Pilbara, Western Australia. Astrobiology.

[5] Schopf J.W., Kudryavtsev A.B., Agresti D.G., Wdowiak T.J., Czaja A.D. (2002). Laser–Raman Imagery of Earth’s Earliest Fossils. Nature.

[6] Brasier M.D., Green O.R., Jephcoat A.P., Kleppe A.K., Van Kranendonk M.J., Lindsay J.F., Steele A., Grassineau N.V. (2002). Questioning the Evidence for Earth’s Oldest Fossils. Nature.

[7] Javaux E.J. (2019). Challenges in Evidencing the Earliest Traces of Life. Nature.

[8] Cady S.L., Farmer J.D., Grotzinger J.P., Schopf J.W., Steele A. (2003). Morphological Biosignatures and the Search for Life on Mars. Astrobiology.

[9] Schopf J.W. (1993). Microfossils of the Early Archean Apex Chert: New Evidence of the Antiquity of Life. Science.

[10] Westall F., Hickman-Lewis K., Cavalazzi B. (2019). Biosignatures in deep time. Biosignatures for Astrobiology.

[11] Hays L.E., Graham H.V., Des Marais D.J., Hausrath E.M., Horgan B., McCollom T.M., Parenteau M.N., Potter-McIntyre S.L., Williams A.J., Lynch K.L. (2017). Biosignature Preservation and Detection in Mars Analog Environments. Astrobiology.

[12] Tarnas J.D., Mustard J.F., Lin H., Goudge T.A., Amador E.S., Bramble M.S., Kremer C.H., Zhang X., Itoh Y., Parente M. (2019). Orbital Identification of Hydrated Silica in Jezero Crater, Mars. Geophys. Res. Lett..

[13] Goudge T.A., Mustard J.F., Head J.W., Fassett C.I., Wiseman S.M. (2015). Assessing the Mineralogy of the Watershed and Fan Deposits of the Jezero Crater Paleolake System, Mars. J. Geophys. Res. Planets.

[14] Osinski G.R., Tornabene L.L., Banerjee N.R., Cockell C.S., Flemming R., Izawa M.R., McCutcheon J., Parnell J., Preston L.J., Pickersgill A.E. (2013). Impact-Generated Hydrothermal Systems on Earth and Mars. Icarus.

[15] Ruff S.W., Farmer J.D. (2016). Silica Deposits on Mars with Features Resembling Hot Spring Biosignatures at El Tatio in Chile. Nat. Commun..

[16] Walter M.R., Des Marais D.J. (1993). Preservation of Biological Information in Thermal Spring Deposits: Developing a Strategy for the Search for Fossil Life on Mars. Icarus.

[17] Cady S.L., Farmer J.D. (1996). Fossilization Processes in Siliceous Thermal Springs: Trends in Preservation along Thermal Gradients. Ciba Found Symp..

[18] Lynne B.Y., Campbell K.A., James B.J., Browne P.R., Moore J. (2007). Tracking Crystallinity in Siliceous Hot-Spring Deposits. Am. J. Sci..

[19] Schopf J.W. (1977). Evidences of Archean Life. Chemical Evolution of the Early Precambrian.

[20] Djokic T., Van Kranendonk M.J., Campbell K.A., Walter M.R., Ward C.R. (2017). Earliest Signs of Life on Land Preserved in ca. 3.5 Ga Hot Spring Deposits. Nat. Commun..

[21] Damer B., Deamer D. (2019). The Hot Spring Hypothesis for an Origin of Life. Astrobiology.

[22] Olgers F. (1972). Geology of the Drummond Basin, Queensland.

[23] Walter M.R., McLoughlin S., Drinnan A.N., Farmer J.D. (1998). Palaeontology of Devonian Thermal Spring Deposits, Drummond Basin, Australia. Alcheringa.

[24] Walter M.R., Desmarais D., Farmer J.D., Hinman N.W. (1996). Lithofacies and Biofacies of Mid-Paleozoic Thermal Spring Deposits in the Drummond Basin, Queensland, Australia. Palaios.

[25] Cunneen R., Sillitoe R.H. (1989). Paleozoic Hot Spring Sinter in the Drummond Basin, Queensland, Australia. Econ. Geol..

[26] Gong J., Myers K.D., Munoz-Saez C., Homann M., Rouillard J., Wirth R., Schreiber A., van Zuilen M.A. (2020). Formation and Preservation of Microbial Palisade Fabric in Silica Deposits from El Tatio, Chile. Astrobiology.

[27] Ball J.W., McMleskey R.B., Nordstrom D.K. (2010). Water-Chemistry Data for Selected Springs, Geysers, and Streams in Yellowstone National Park, Wyoming, 2006-2008.

[28] Gangidine A., Havig J.R., Fike D.A., Jones C., Hamilton T.L., Czaja A.D. (2020). Trace Element Concentrations in Hydrothermal Silica Deposits as a Potential Biosignature. Astrobiology.

[29] Havig J.R. (2009). Geochemistry of Hydrothermal Biofilms: Composition of Biofilms in a Siliceous Sinter-Deposition Hot Spring.

[30] Martin R., Rodgers K.A., Browne P.R.L. (2000). Aspects of the Distribution and Movement of Aluminium in the Surface of the Te Kopia Geothermal Field, Taupo Volcanic Zone, New Zealand. Appl. Geochem..

[31] Banfield J.F., Moreau J.W., Chan C.S., Welch S.A., Little B. (2001). Mineralogical Biosignatures and the Search for Life on Mars. Astrobiology.

[32] Webb G.E., Kamber B.S. (2011). Trace element geochemistry as a tool for interpreting microbialites. Earliest Life on Earth: Habitats, Environments and Methods of Detection.

[33] Heim C., Simon K., Ionescu D., Reimer A., De Beer D., Quéric N.-V., Reitner J., Thiel V. (2015). Assessing the Utility of Trace and Rare Earth Elements as Biosignatures in Microbial Iron Oxyhydroxides. Front. Earth Sci..

[34] Simões M.F., Ottoni C.A., Antunes A. (2020). Biogenic Metal Nanoparticles: A New Approach to Detect Life on Mars?. Life.

[35] Hickman-Lewis K., Cavalazzi B., Sorieul S., Gautret P., Foucher F., Whitehouse M.J., Jeon H., Georgelin T., Cockell C.S., Westall F. (2020). Metallomics in Deep Time and the Influence of Ocean Chemistry on the Metabolic Landscapes of Earth’s Earliest Ecosystems. Sci. Rep..

[36] Allwood A., Clark B., Flannery D., Hurowitz J., Wade L., Elam T., Foote M., Knowles E. Texture-Specific Elemental Analysis of Rocks and Soils with PIXL: The Planetary Instrument for X-Ray Lithochemistry on Mars 2020. Proceedings of the 2015 IEEE Aerospace Conference.

[37] Sforna M.-C., Daye M., Philippot P., Somogyi A., Van Zuilen M.A., Medjoubi K., Gérard E., Jamme F., Dupraz C., Braissant O. (2017). Patterns of Metal Distribution in Hypersaline Microbialites during Early Diagenesis: Implications for the Fossil Record. Geobiology.

[38] Korotev R.L., Zeigler R.A., Jolliff B.L., Irvin A.J., Bunch T.E. (2009). Compositional and Lithological Diversity among Brecciated Lunar Meteorites of Intermediate Iron Concentration. Meteorit. Planet. Sci..

[39] Wilson R.G. (1988). Secondary Ion Mass Spectrometry Sensitivity Factors versus Ionization Potential and Electron Affinity for Many Elements in HgCdTe and CdTe Using Oxygen and Cesium Ion Beams. J. Appl. Phys..

[40] Johnson D.B. (2006). Biohydrometallurgy and the Environment: Intimate and Important Interplay. Hydrometallurgy.

[41] Lynne B.Y., Campbell K.A., Moore J.N., Browne P.R.L. (2005). Diagenesis of 1900-Year-Old Siliceous Sinter (Opal-A to Quartz) at Opal Mound, Roosevelt Hot Springs, Utah, USA. Sediment. Geol..

[42] Hickman-Lewis K., Gourcerol B., Westall F., Manzini D., Cavalazzi B. (2020). Reconstructing Palaeoarchaean Microbial Biomes Flourishing in the Presence of Emergent Landmasses Using Trace and Rare Earth Element Systematics. Precambrian Res..

[43] Huerta-Diaz M.A., Delgadillo-Hinojosa F., Siqueiros-Valencia A., Valdivieso-Ojeda J., Reimer J.J., Segovia-Zavala J.A. (2012). Millimeter-scale Resolution of Trace Metal Distributions in Microbial Mats from a Hypersaline Environment in Baja California, Mexico. Geobiology.

[44] Allwood A.C., Kamber B.S., Walter M.R., Burch I.W., Kanik I. (2010). Trace Elements Record Depositional History of an Early Archean Stromatolitic Carbonate Platform. Chem. Geol..

