# Supplementary material for: Trace Element Concentrations Associated with Mid-Paleozoic Microfossils as Biosignatures to Aid in the Search for Life
Source: Life (Basel). 2021 Feb 13;11(2):142. doi: 10.3390/life11020142 (PMC7918189; doi:10.3390/life11020142)
Supplement: Supplementary file 1 [file life-11-00142-s001.pdf]

Supplementary Material

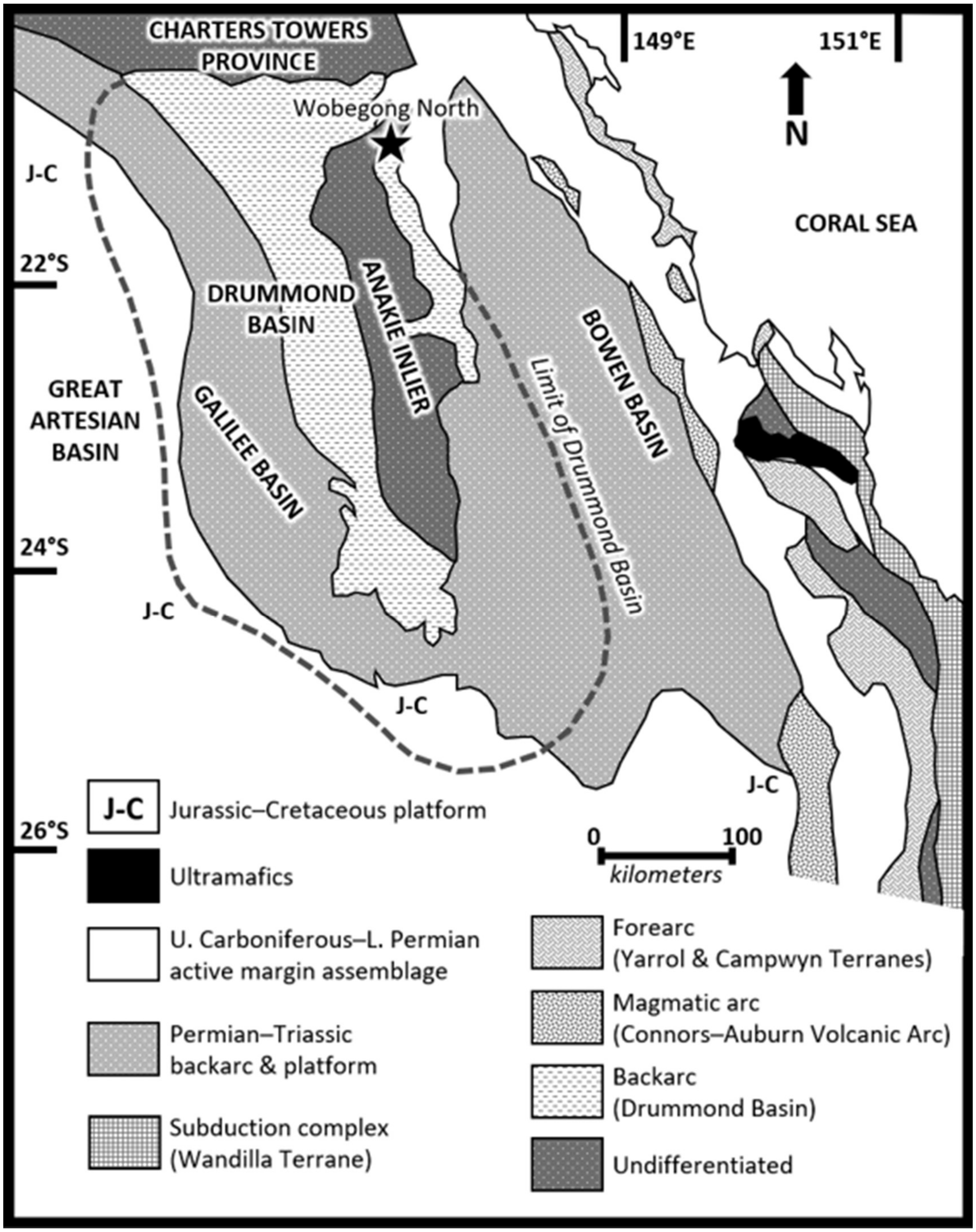

Figure S1. Geologic map of Drummond Basin, showing the location of the Wobegong North sinters within the Conway Hydrothermal System, where the samples from this study were collected (modified from Walter et al. 1996).

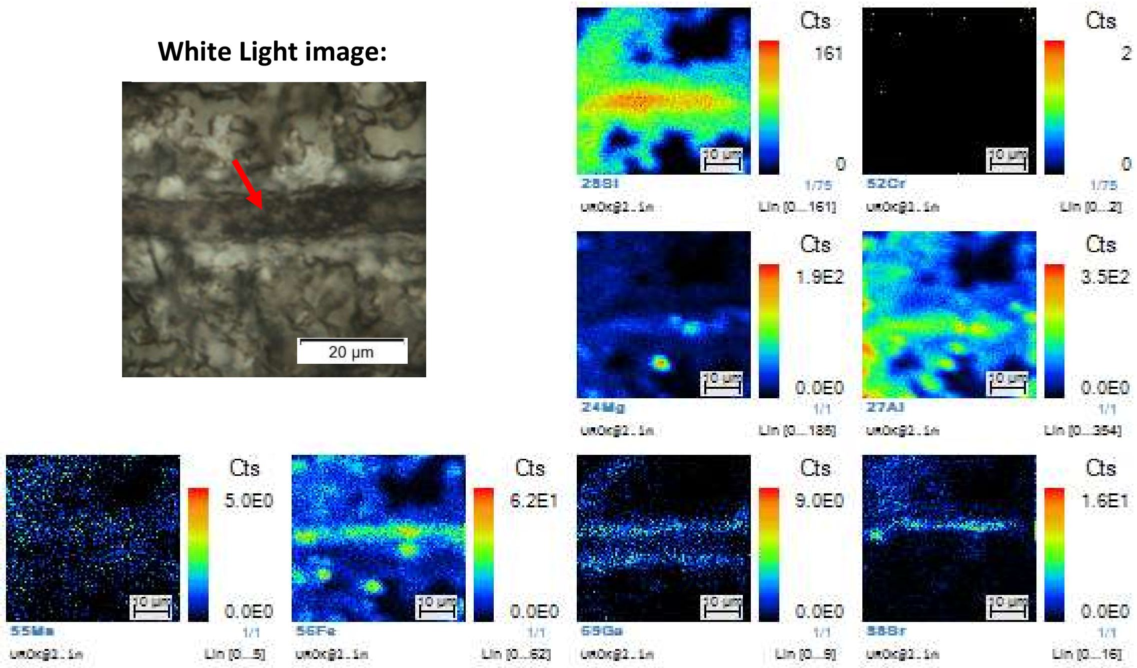

Figure S2. SIMS analyses of a <10,000-year-old cyanobacterial microorganism (noted by red arrow) preserved in a hot spring deposit in Yellowstone National Park, showing similar elemental sequestration trends (notably Sr, Fe, Mg) as to what was observed in Drummond samples (see also Gangidine et al. 2020), but also displaying unique trends (e.g., Gallium).

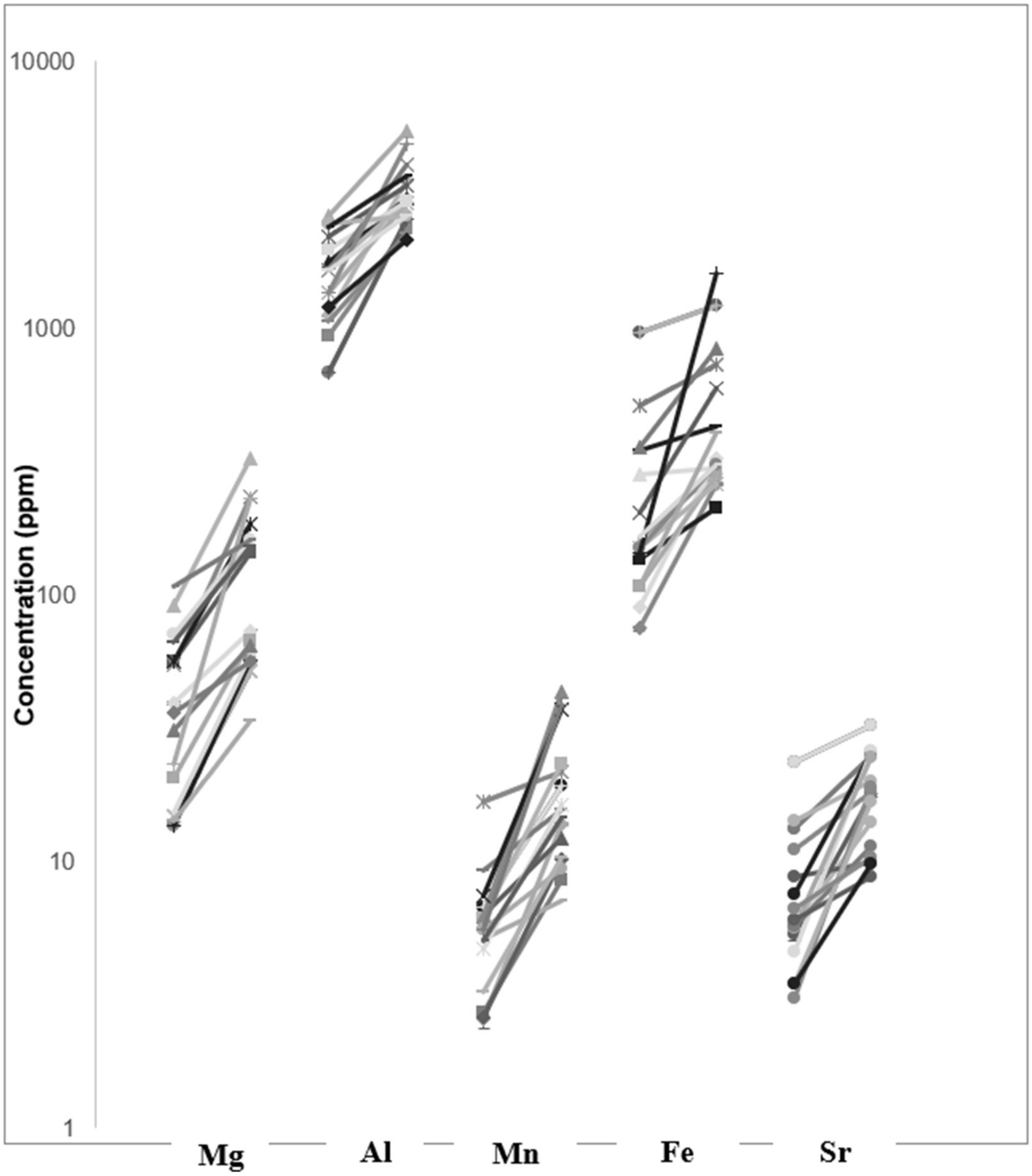

Figure S3. Visual graph illustrating the same data as Figure 3 in the main text, with each background value (left point) attached to the fossil concentration (right point) to show the increased concentration in each fossil analyzed.

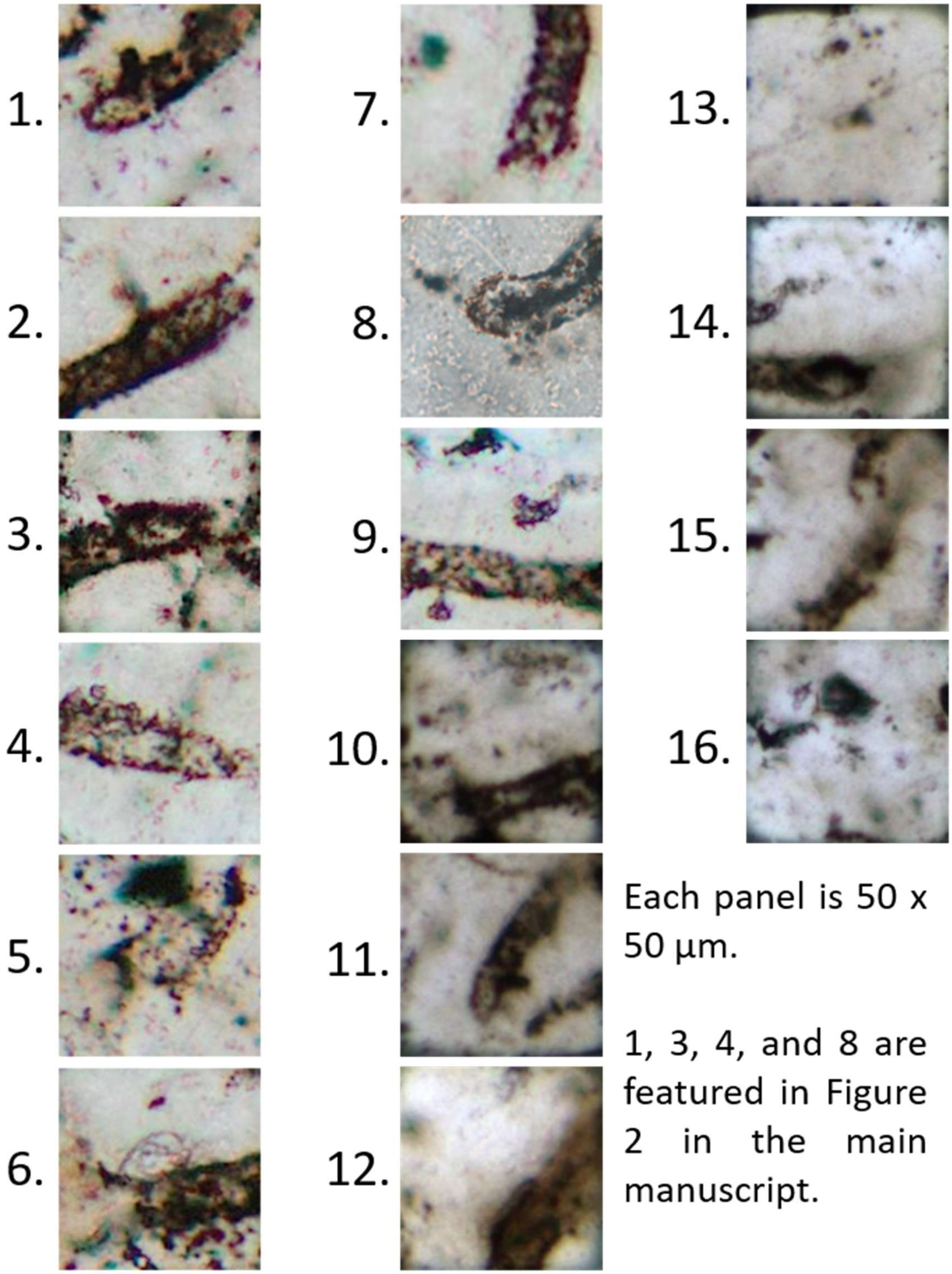

Each panel is 50 x 50 µm.

1, 3, 4, and 8 are featured in Figure 2 in the main manuscript.

Figure S4 (1–16). The above photomicrographs show the 16 Drummond Basin microfossils analyzed in this study. The following pages show the SIMS images generated for each microfossil. Each panel shows a greyscale SIMS image for the noted element.

Sample 1

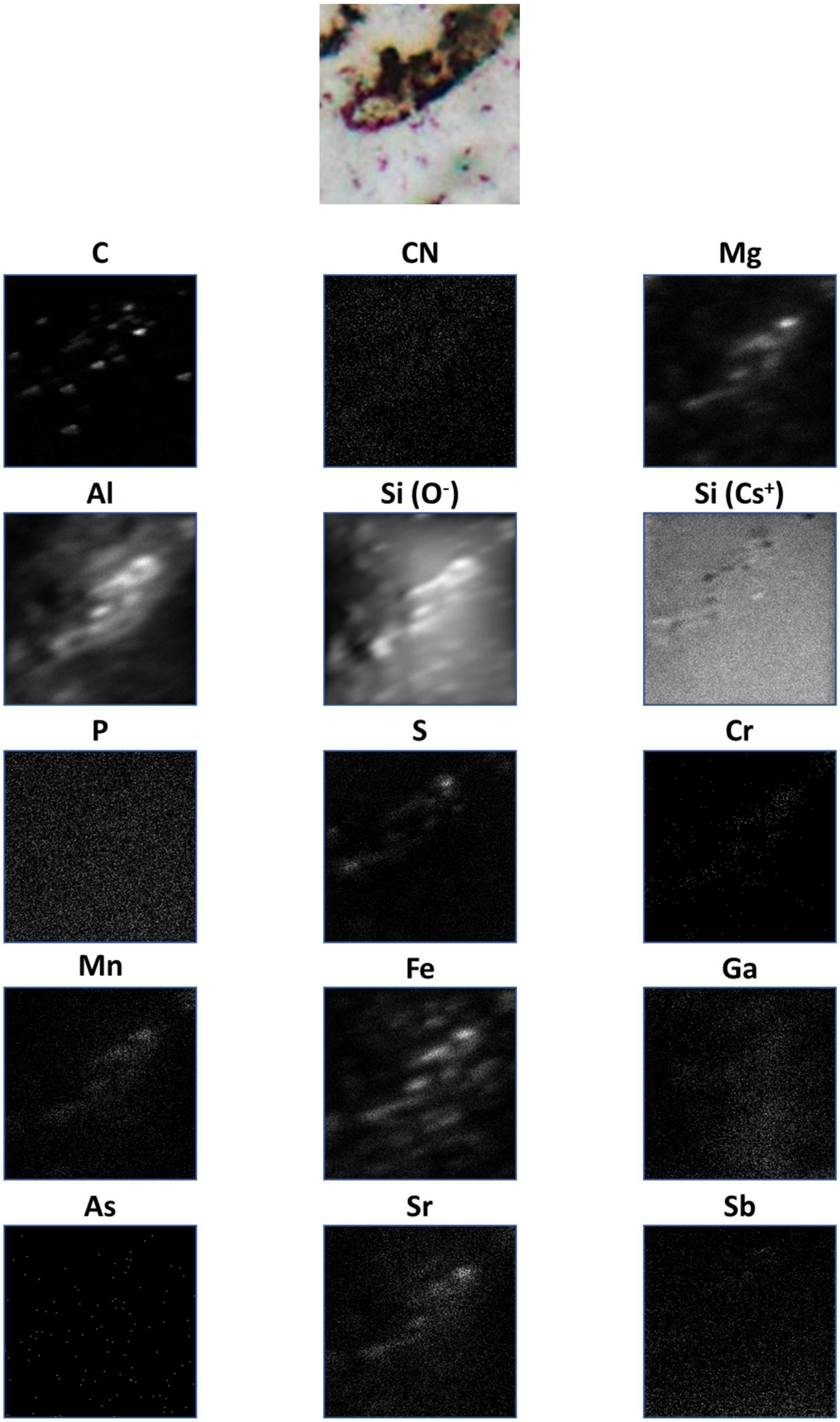

Sample 2

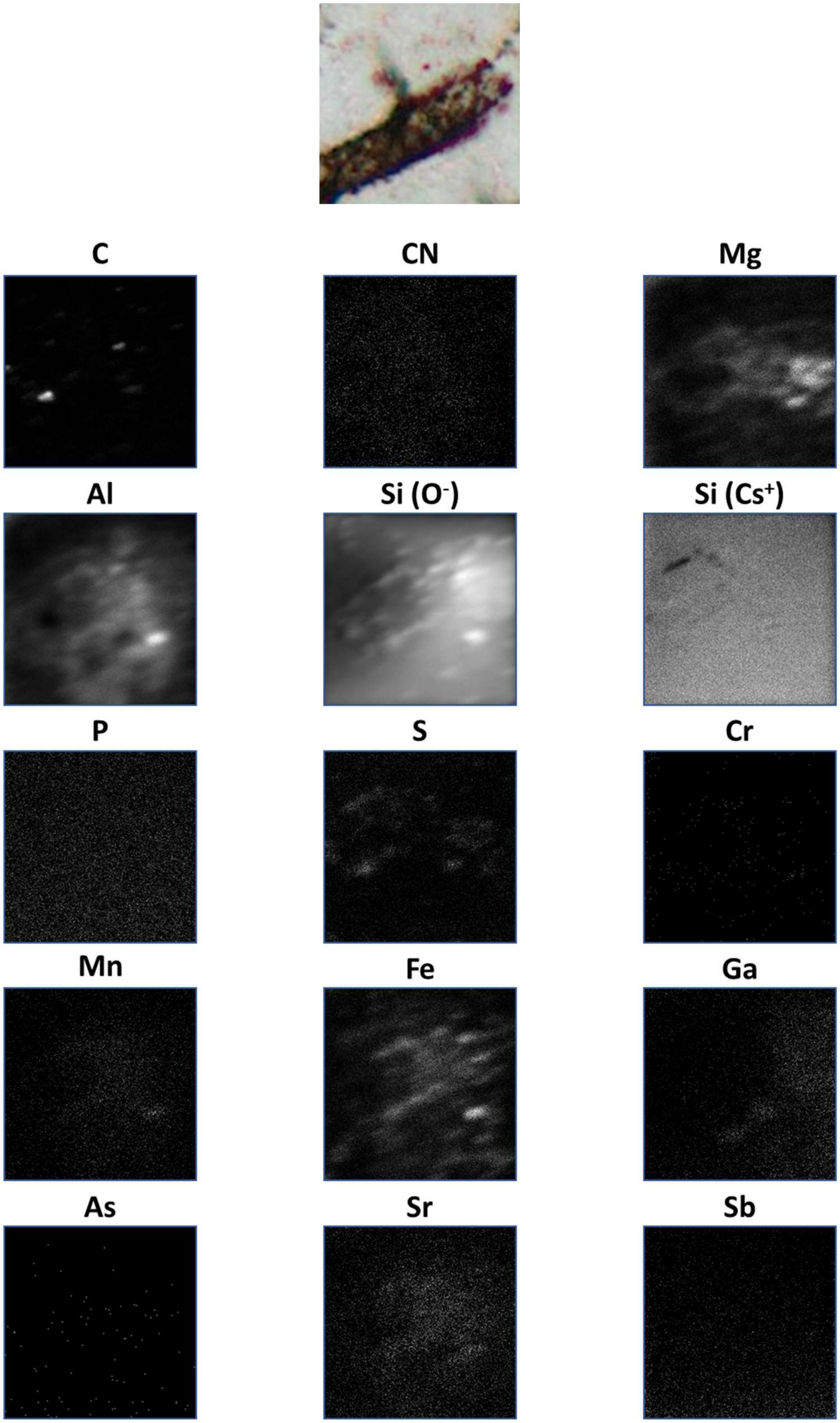

Sample 3

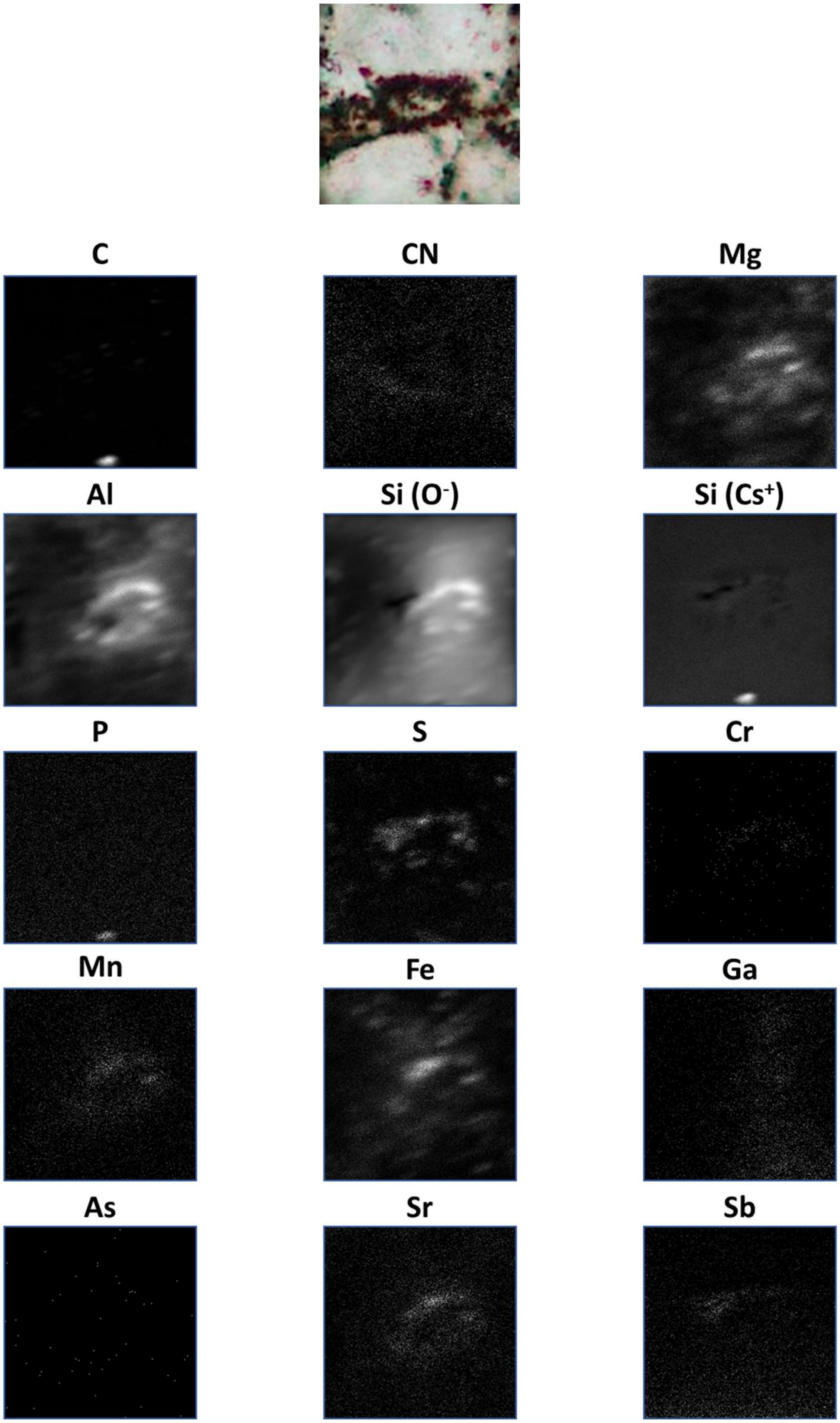

Sample 4

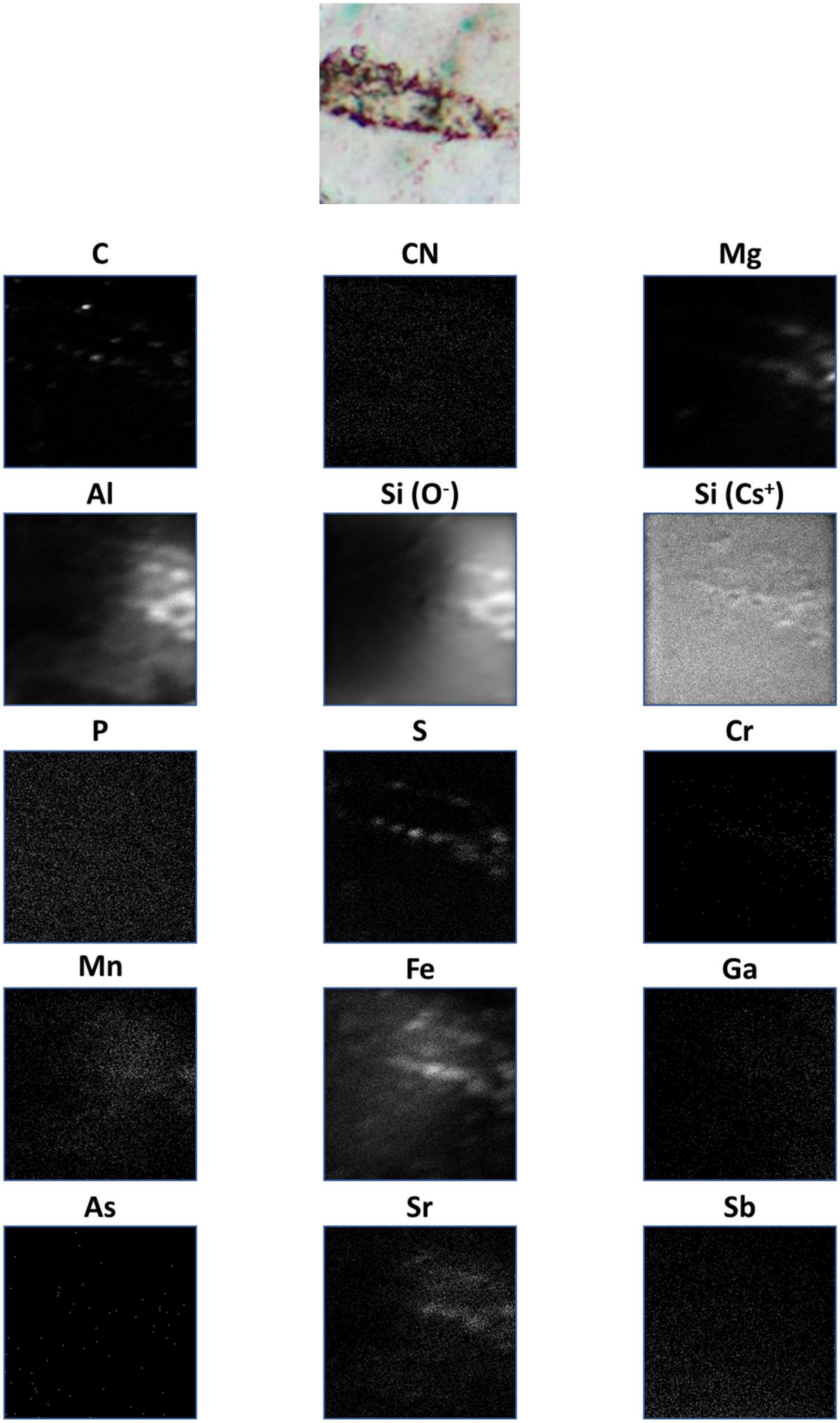

Sample 5

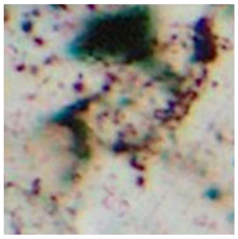

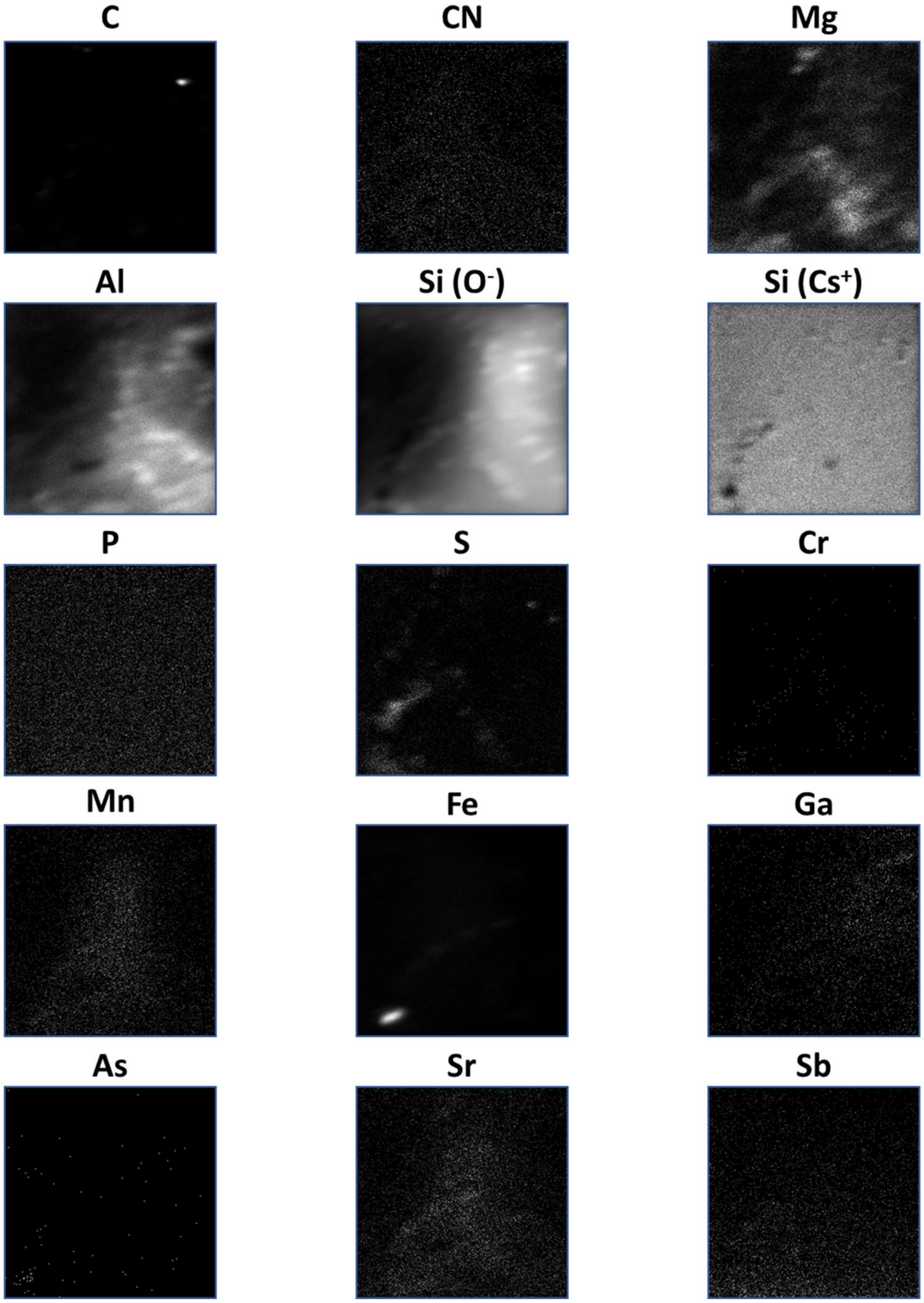

Sample 6

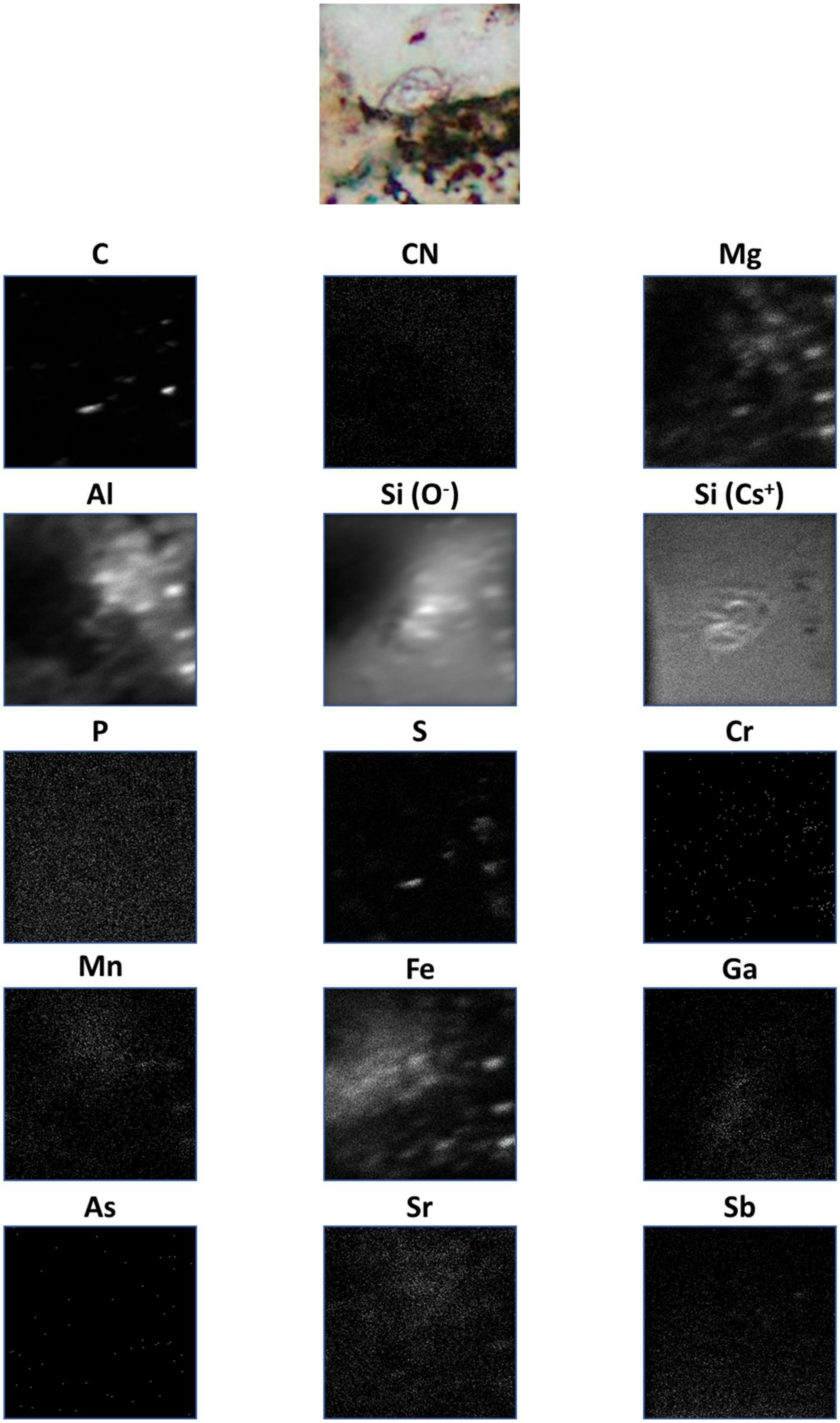

Sample 7

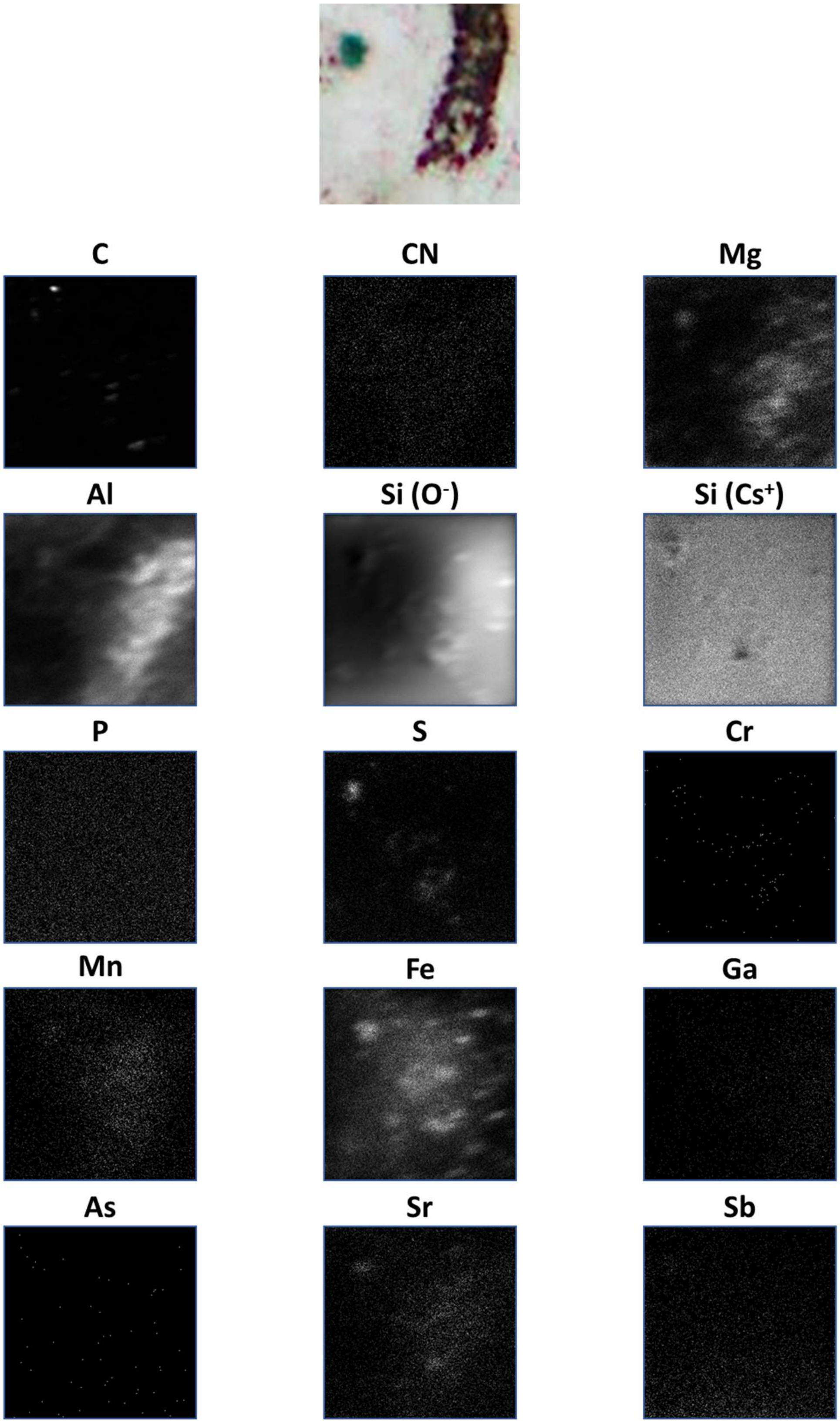

Sample 8

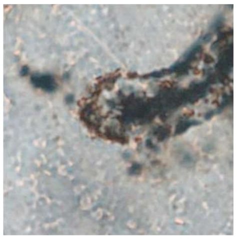

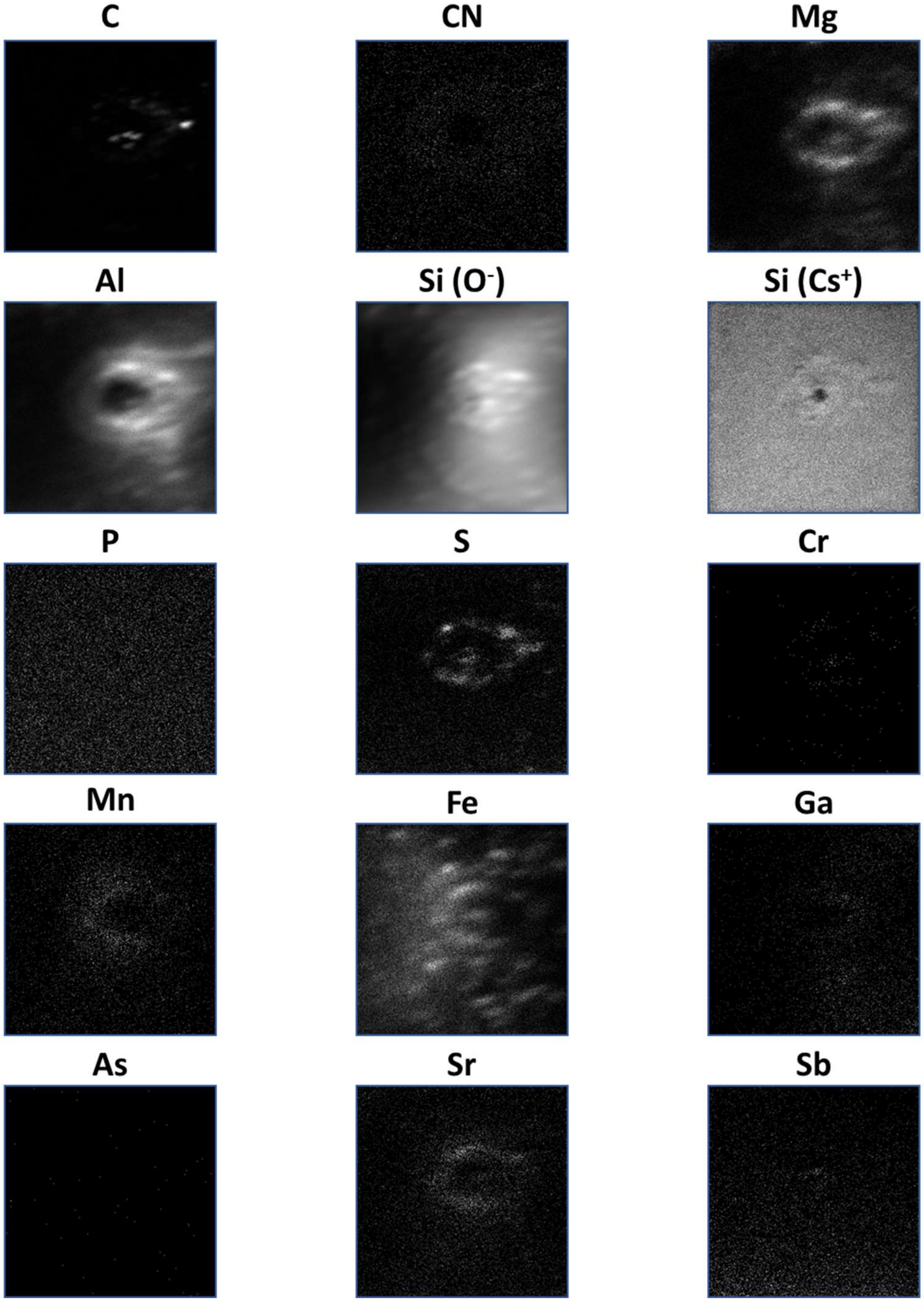

Sample 9

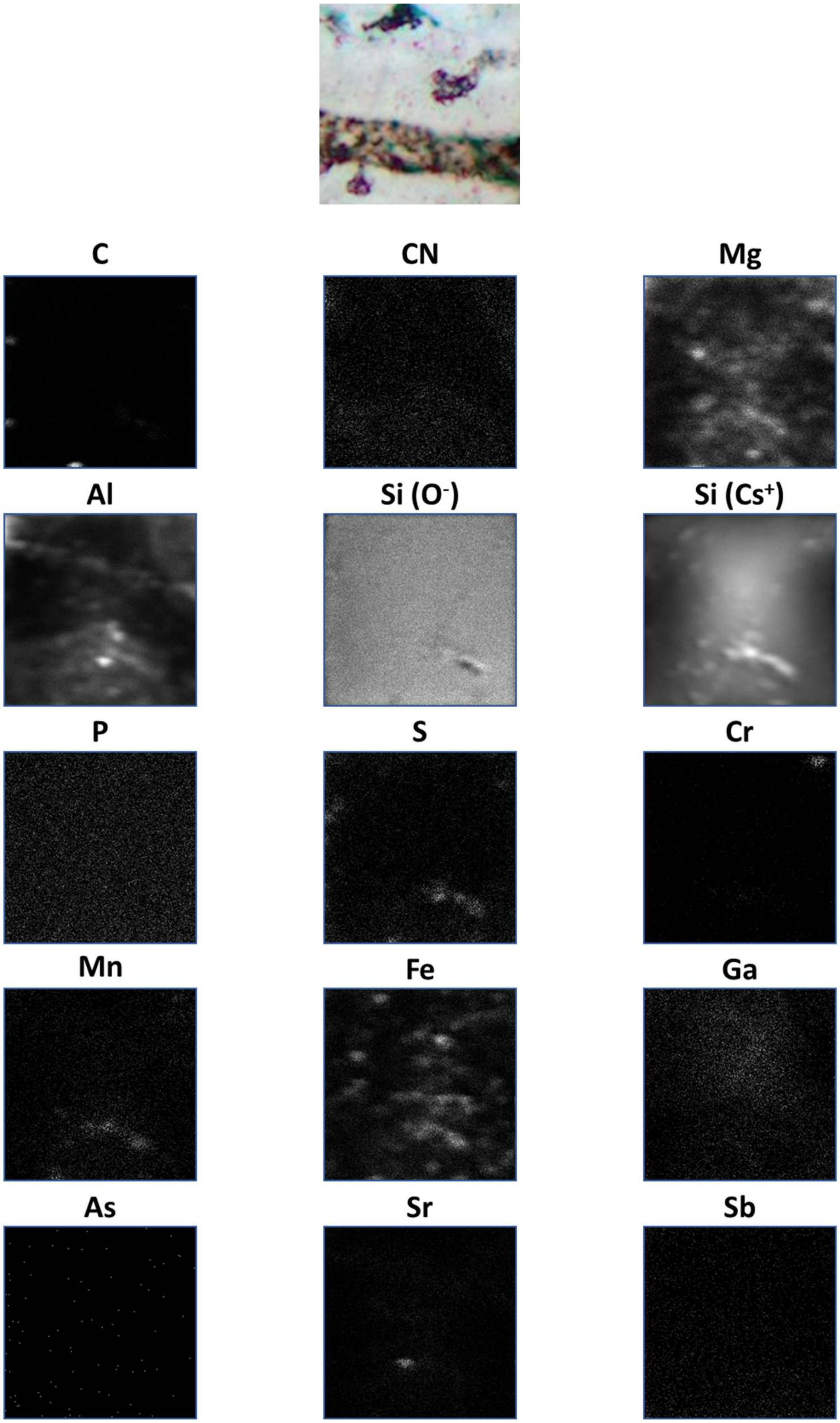

Sample 10

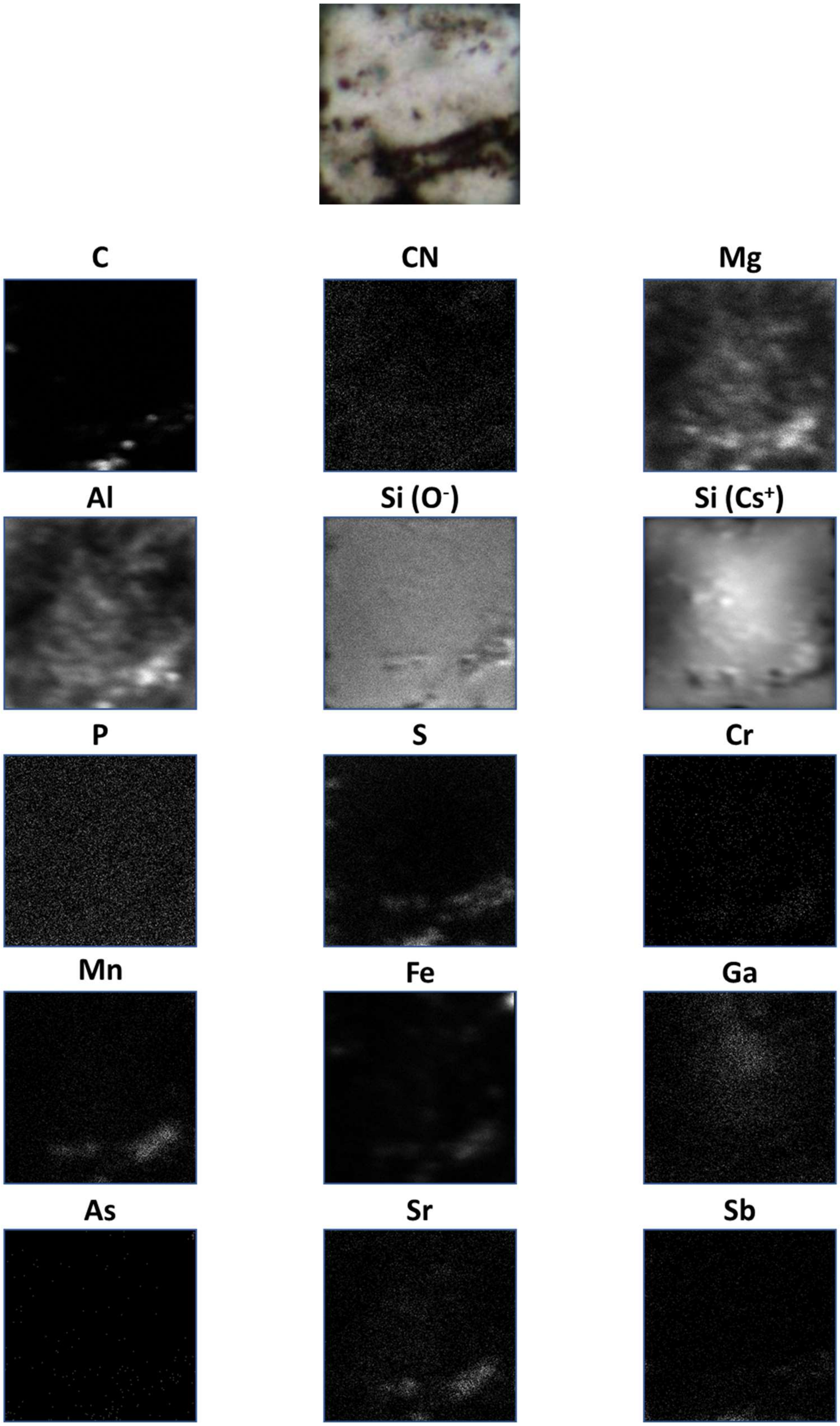

Sample 11

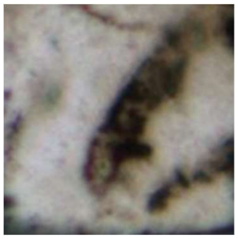

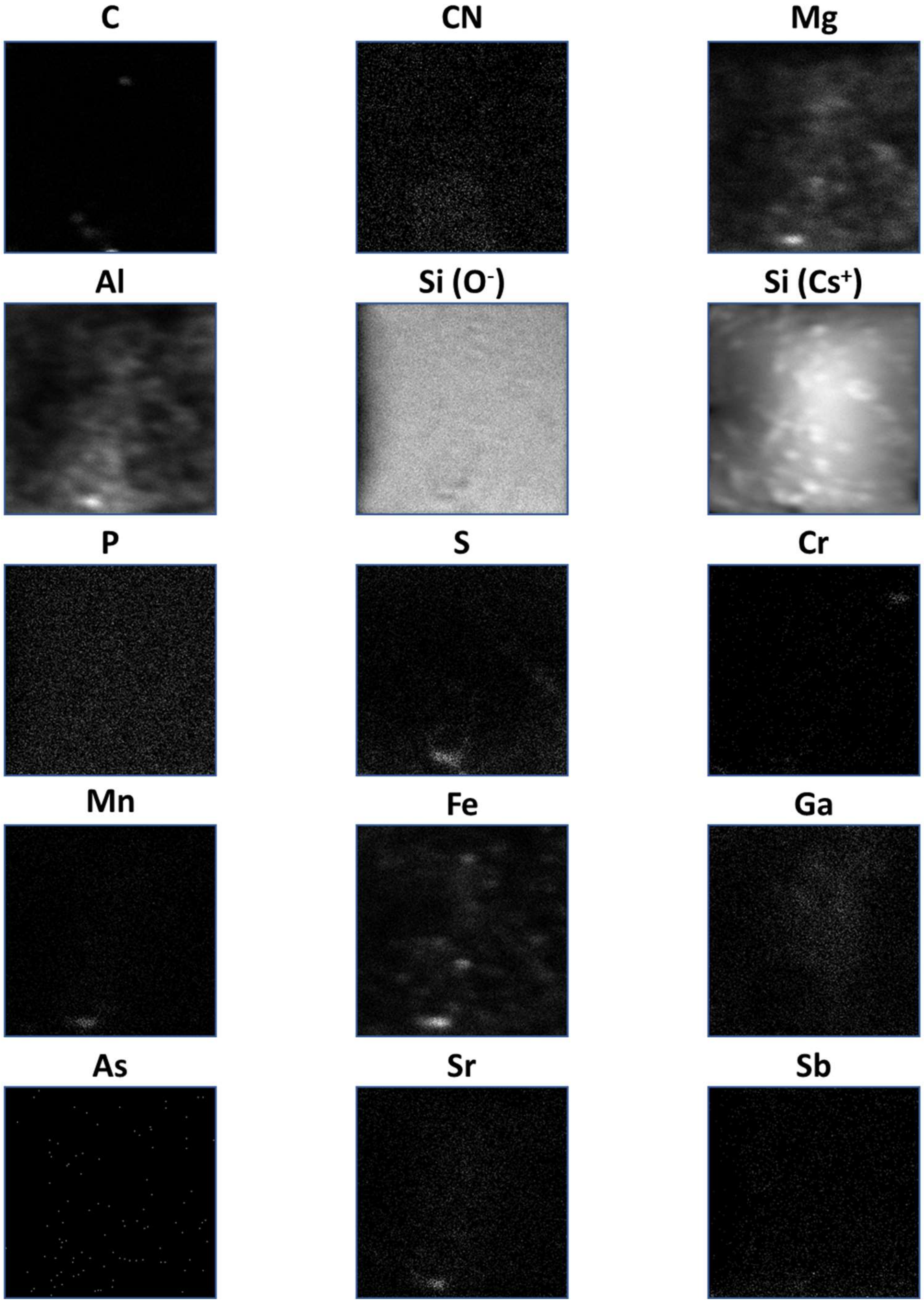

Sample 12

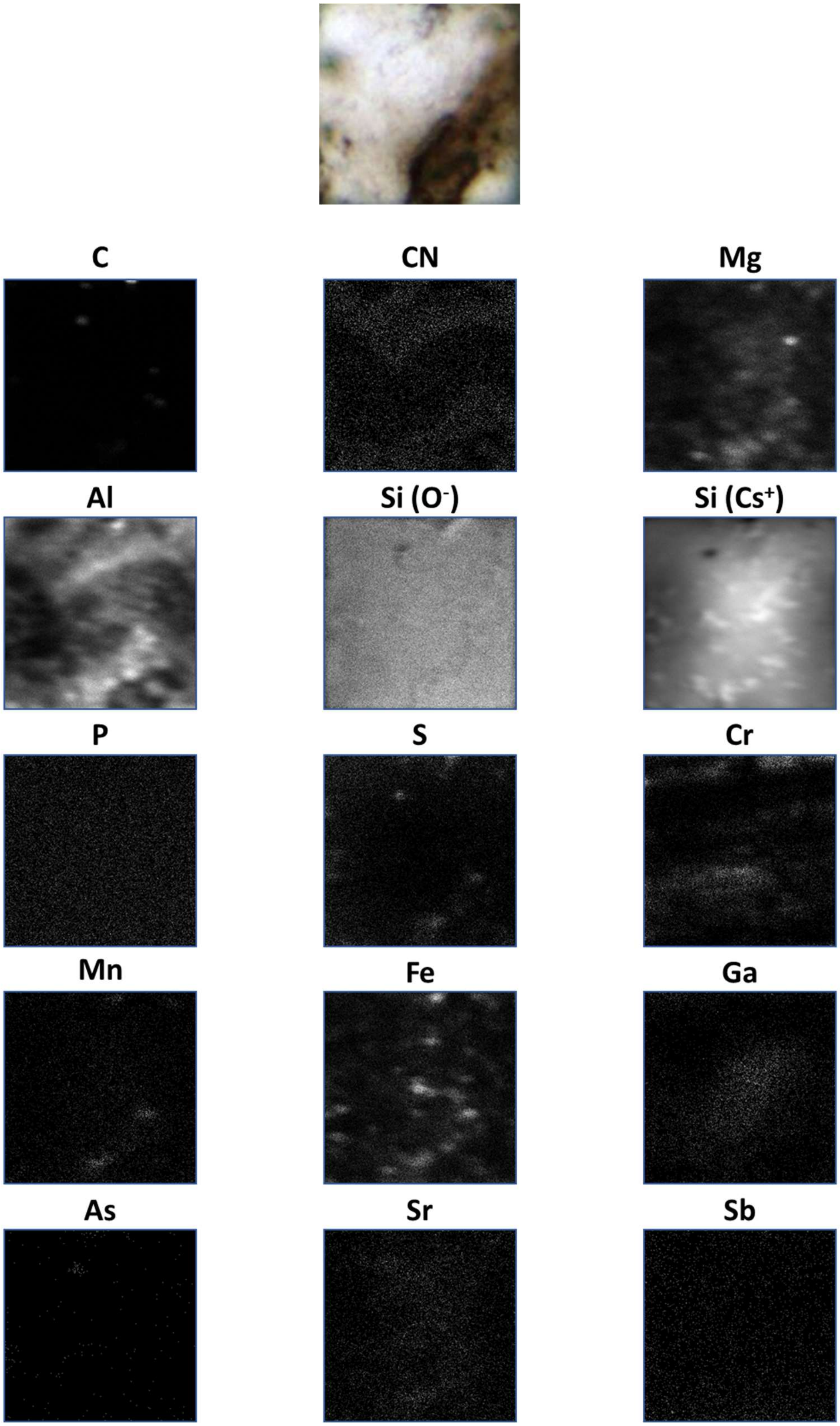

Sample 13

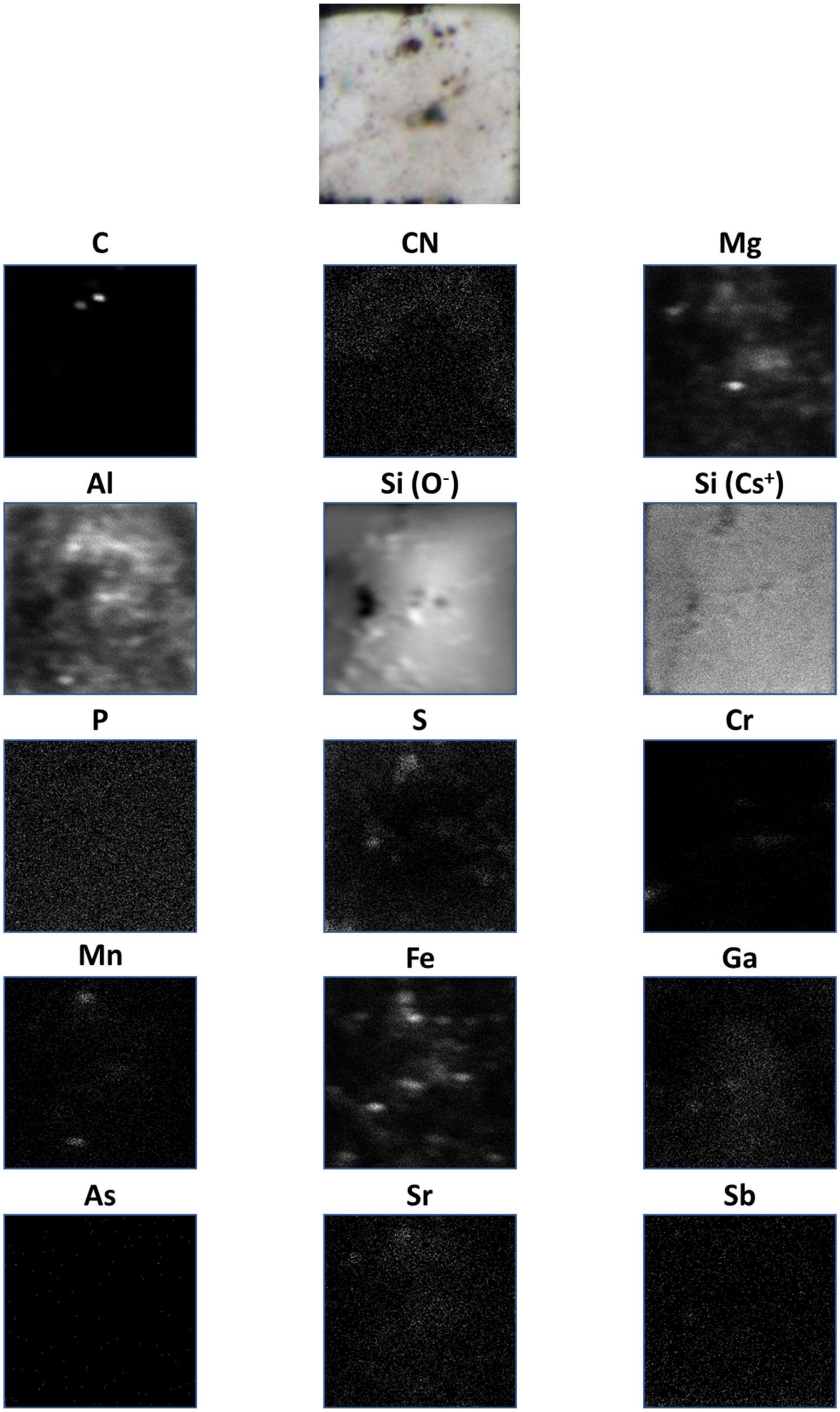

Sample 14

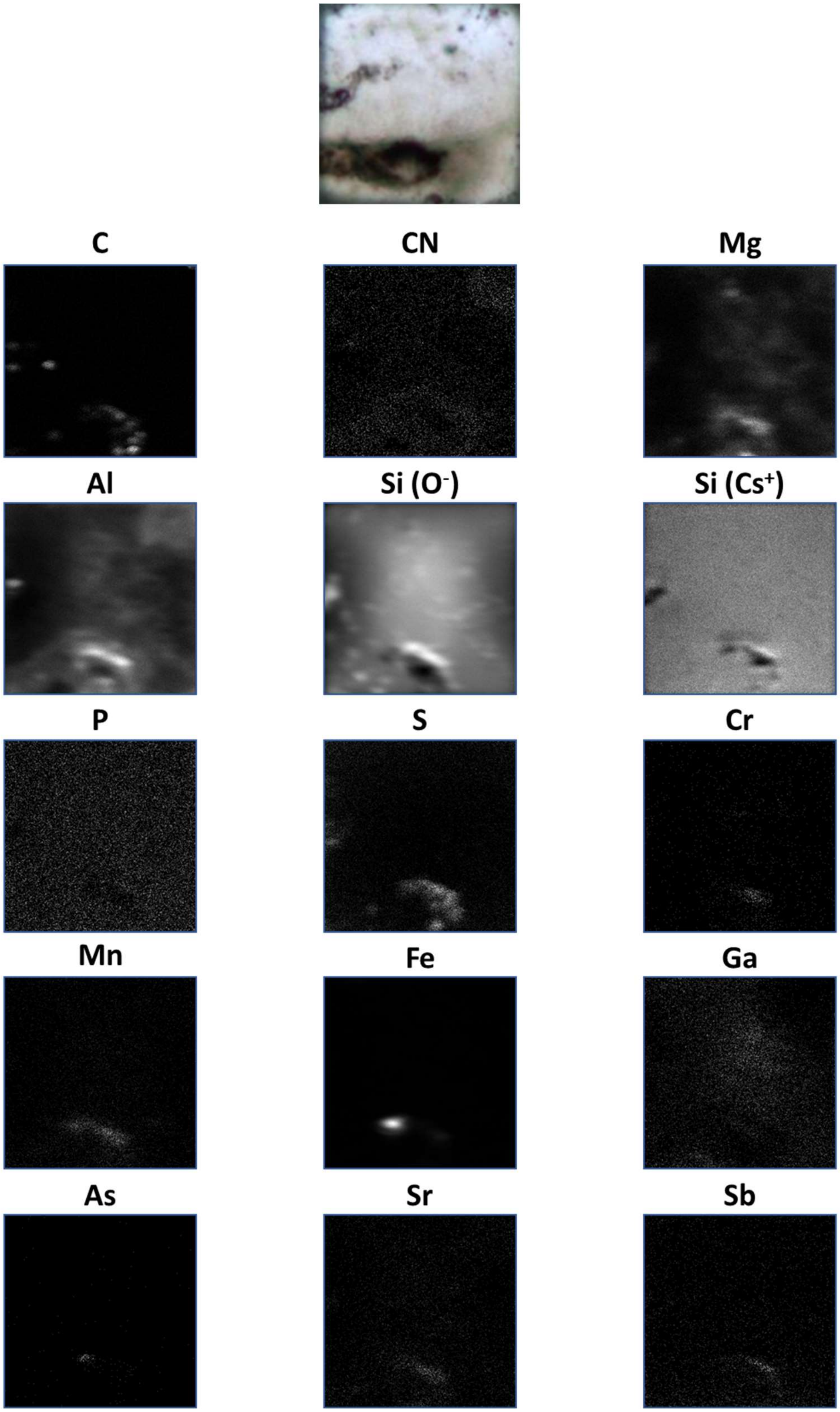

Sample 15

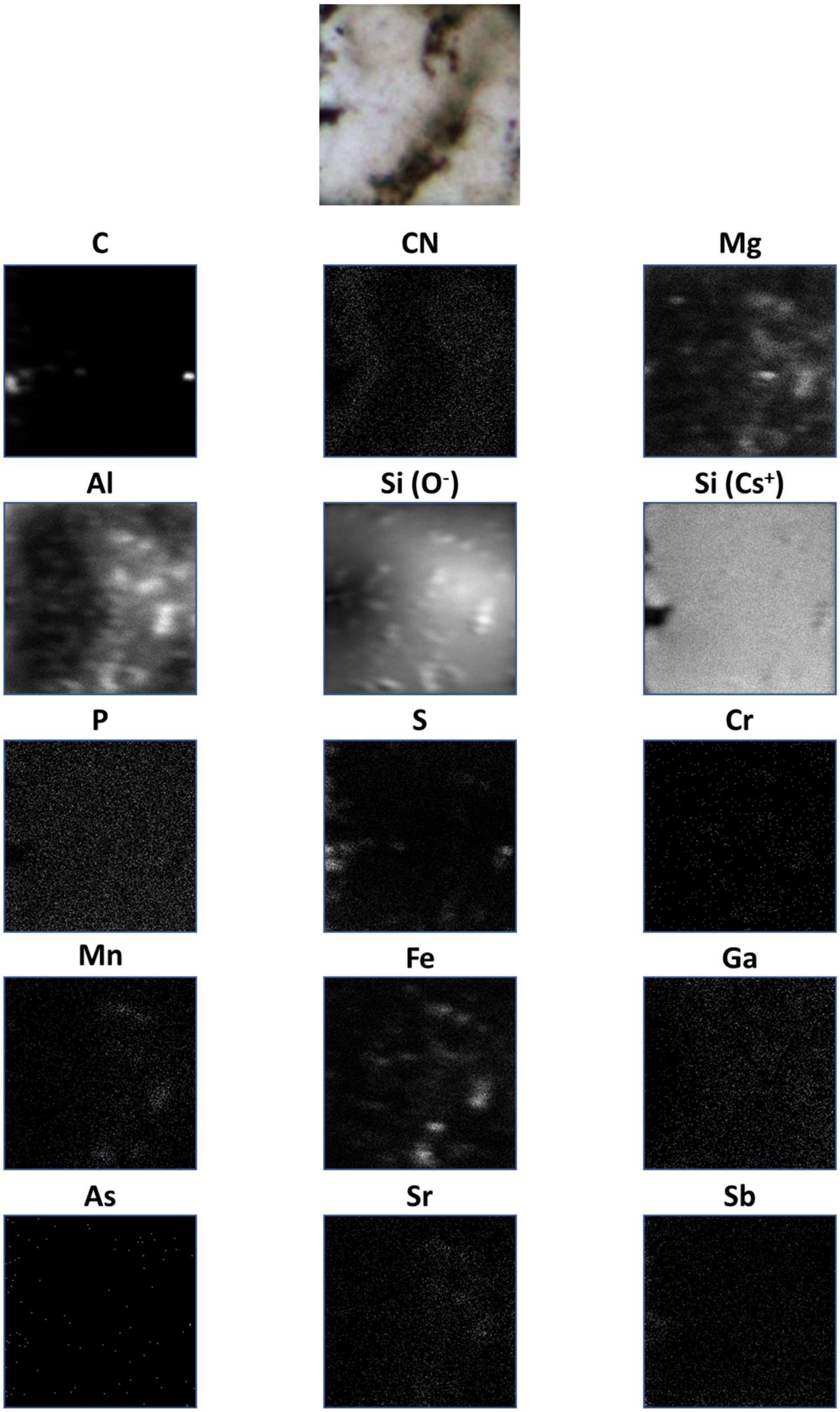

Sample 16

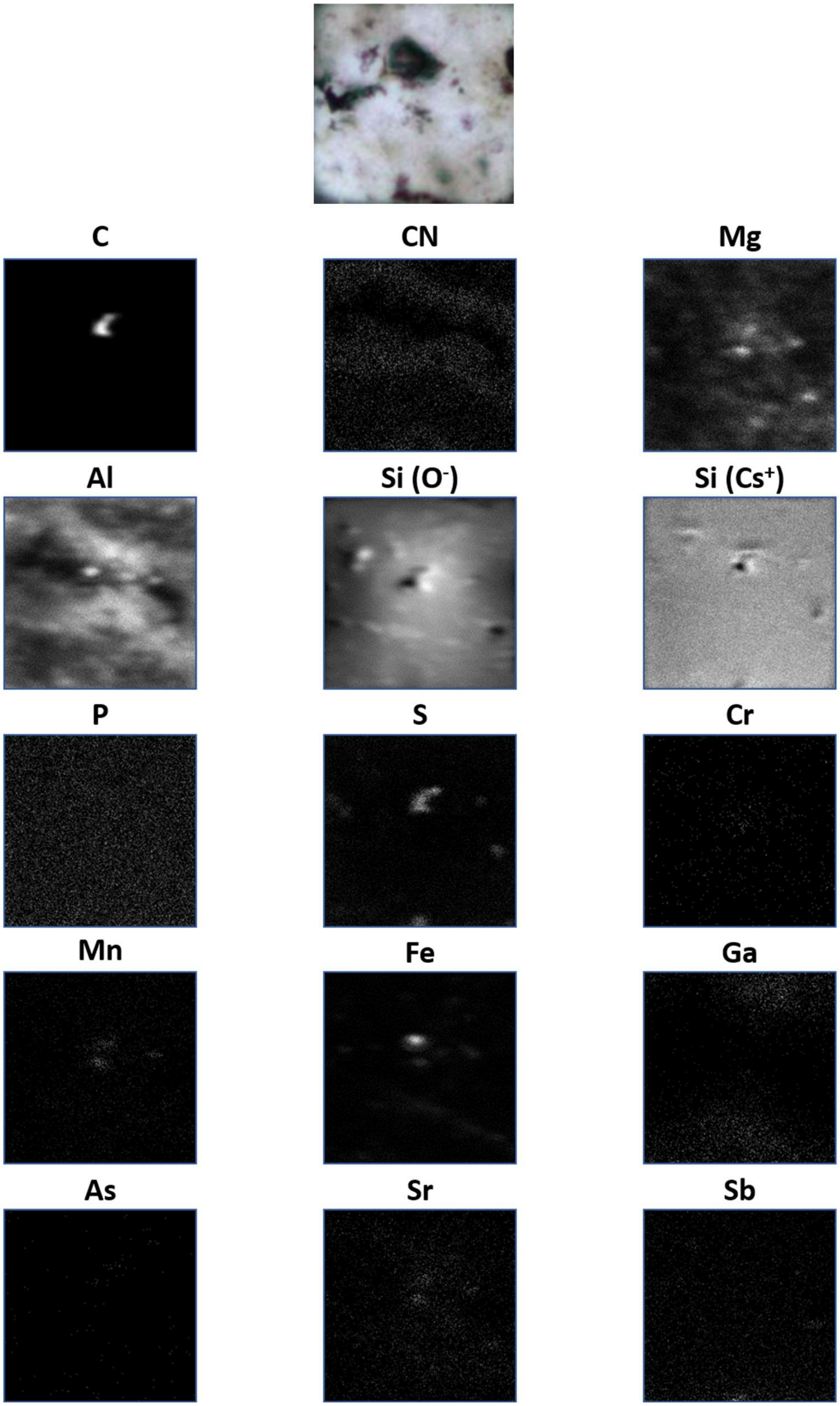

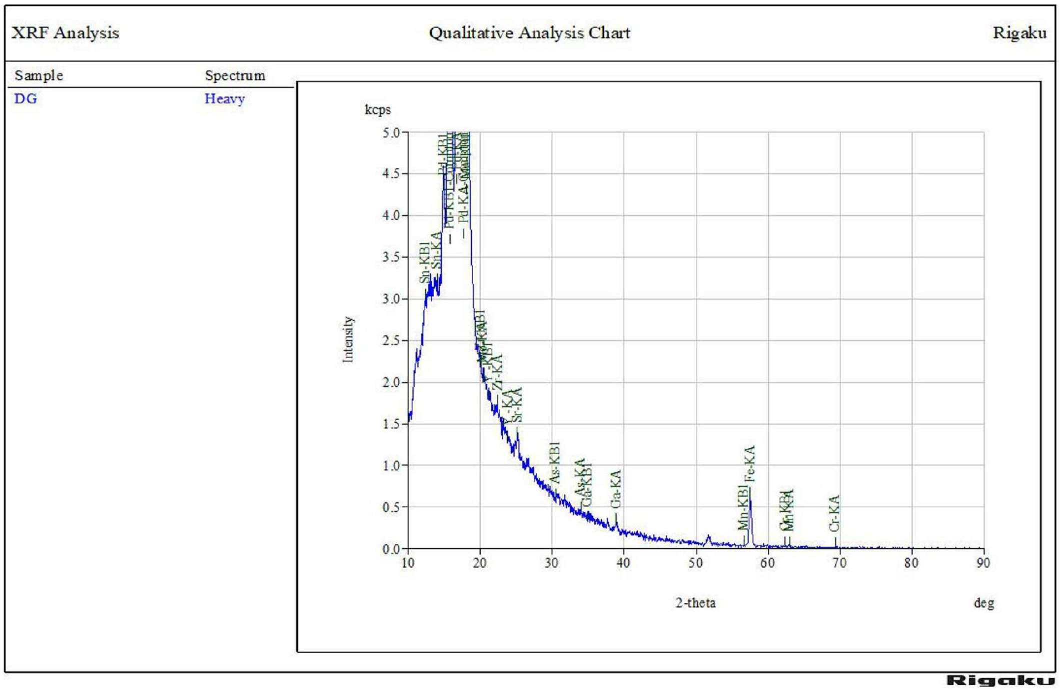

Figure S5. XRF Heavy spectrum from Drummond Basin sample corresponding with Table 1 in the main text.

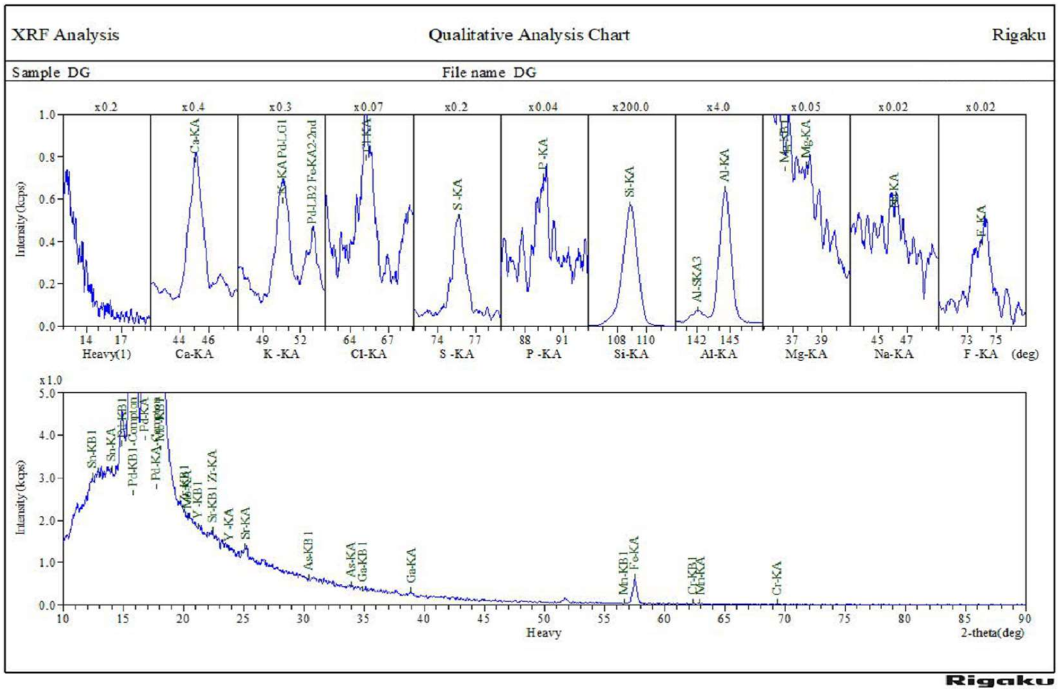

Figure S6. XRF Total spectra from Drummond Basin sample corresponding with Table 1 in the main text.

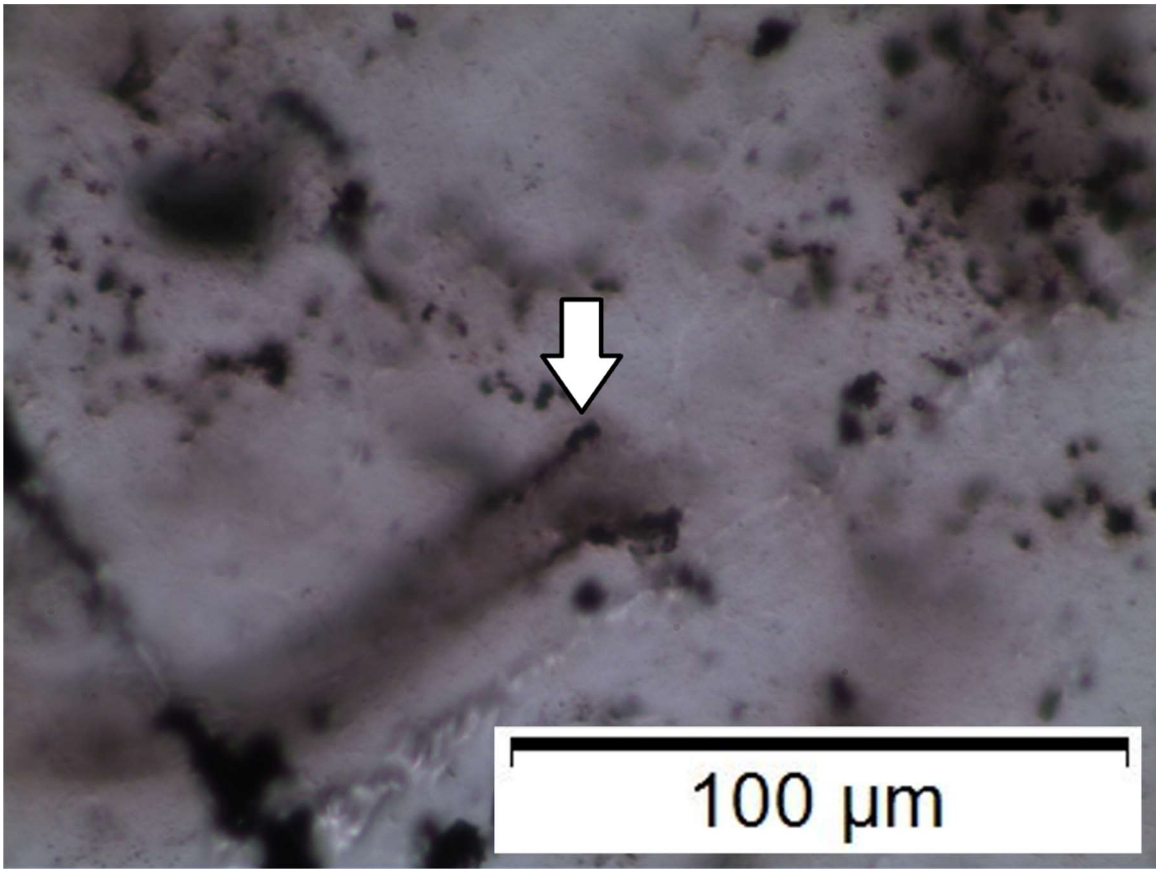

Figure S7. Drummond microfossil targeted by Raman analyses.

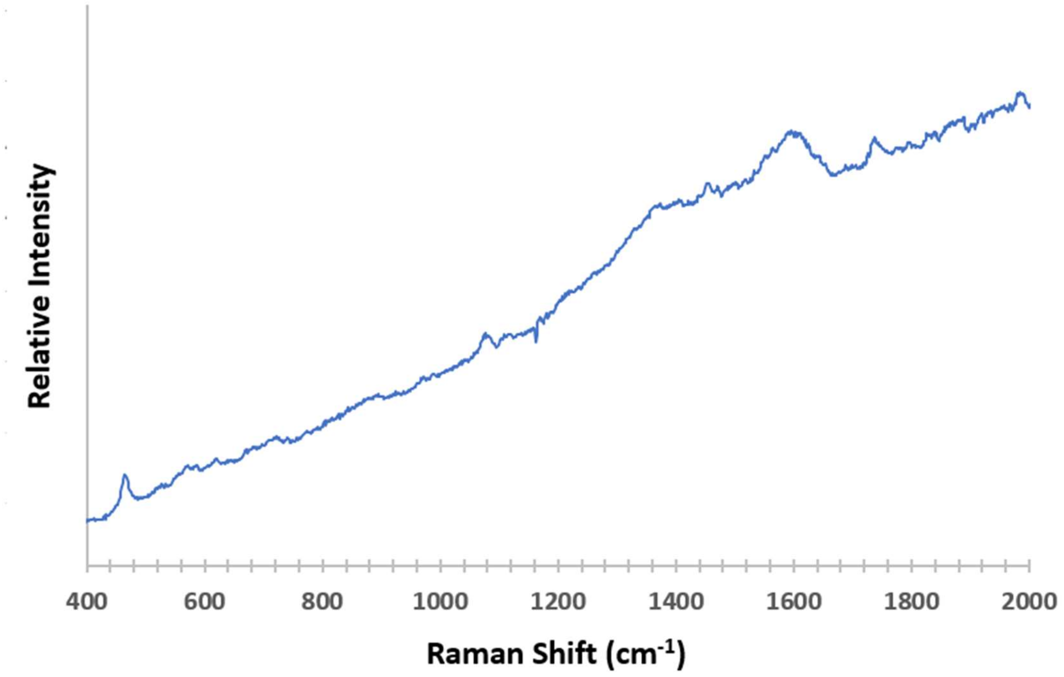

Figure S8. Non-baseline subtracted Raman spectrum from Drummond microfossil body (pictured in Figure S7), showing strong fluorescence.

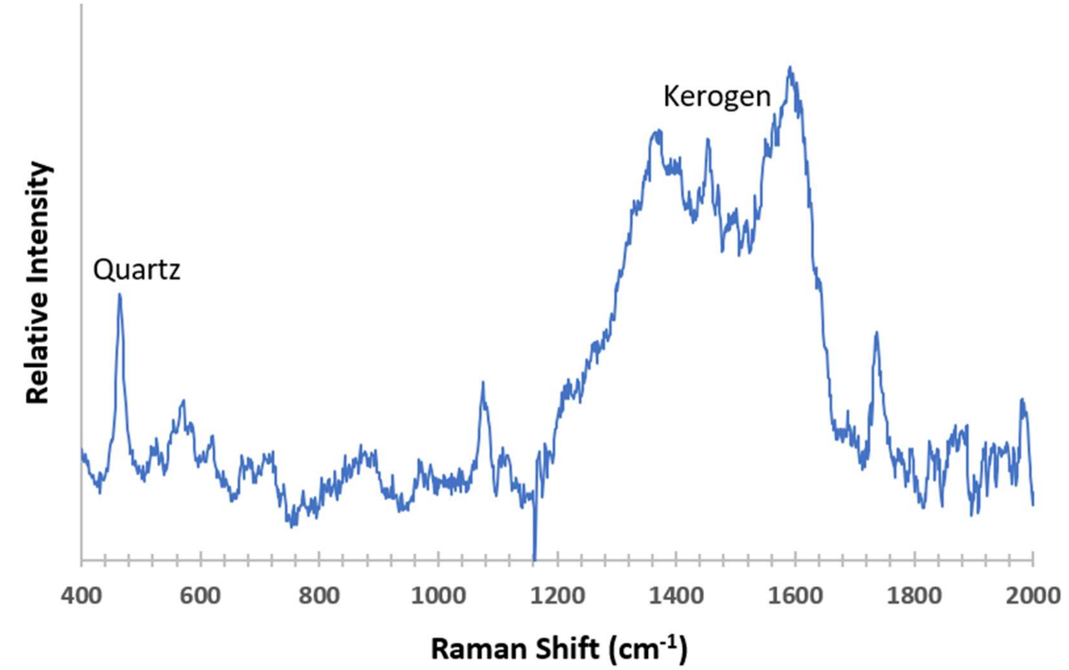

Figure S9. Baseline subtracted Raman spectrum from Figure S8, showing quartz and kerogen. Smaller peaks are due to fluorescence.

Table S1			
**Target Mass**	**Interference**	**MRP* needed**	**Comment**
$C^{15}N$	$^{13}C^{14}N$	4272	does not matter as it is still CN
As	$^{46}Ca^{29}Si$	8722	2 minor isotopes
As	$^{48}Ca^{27}Al$	6004	^{48}Ca minor isotope
As	$^{44}Ca^{30}SiH$	4838	trimers rare, ^{30}Si,^{44}Ca minor
As	$^{46}Ca^{28}SiH$	4447	trimers rare, ^{46}Ca minor
^{120}Sn	$^{48}Ca^{44}Ca^{28}Si$	6949	trimers rare, ^{48},^{45}Ca minor
^{120}Sn	$^{40}Ca^{3}$	8311	trimers rare
^{120}Sn	$^{48}Ca^{44}Ca^{29}Si$	6261	trimers rare, all minor isotopes
^{69}Ga	$^{40}Ca^{29}Si$	5103	^{29}Si minor
^{69}Ga	$^{42}Ca^{27}Al$	4727	^{42}Ca minor
^{69}Ga	$^{46}Ca^{23}Na$	3855	^{46}Ca minor
^{88}Sr	$^{44}Ca^{2}$	16447	^{44}Ca minor
^{88}Sr	$^{48}Ca^{40}Ca$	9255	^{48}Ca minor
^{88}Sr	$^{44}Ca^{43}CaH$	5342	trimers rare, ^{44}Ca ^{43}Ca minor
^{88}Sr	$^{30}Si^{228}Si$	4664	trimers rare, ^{30}Si minor
^{88}Sr	$^{30}Si^{29}Si^{2}$	4158	trimers rare, ^{30}Si 29Si minor
^{88}Sr	$^{42}Ca^{30}Si^{16}O$	4054	trimers rare, ^{42}Ca 30Si minor
^{88}Sr	$^{44}Ca^{28}Si^{16}O$	4050	trimers rare, ^{44}Ca minor
^{88}Sr	$^{46}Ca^{30}Si^{12}C$	4026	trimers rare, ^{46}Ca ^{30}Si minor
^{88}Sr	$^{48}Ca^{28}Si^{12}C$	3688	trimers rare, ^{48}Ca minor
^{88}Sr	$^{43}Ca^{29}Si^{16}O$	3580	trimers rare, ^{43}Ca ^{29}Si minor
^{88}Sr	$^{44}Ca^{29}Si^{15}N$	3322	trimers rare, all minor
^{88}Sr	$^{44}Ca^{20}Si^{14}N$	3292	trimers rare, ^{44}Ca ^{30}Si minor
^{88}Sr	$^{46}Ca^{28}Si^{14}N$	3132	trimers rare, ^{46}Ca minor

Table S1. SIMS interferences and comments for various masses.

Table S2. Elemental data for 16 fossil samples and the surrounding mineral matrix (background) plotted in Figure 3 of the main text.

	Mg				Al				Mn				Fe				Sr			
	Fossil[a]		Background[b]		Fossil		Background		Fossil		Background		Fossil		Background		Fossil		Background	
Sample	Conc.[c]	Err.[d]	Conc.	Err.	Conc.	Err.	Conc.	Err.	Conc.	Err.	Conc.	Err.	Conc.	Err.	Conc.	Err.	Conc.	Err.	Conc.	Err.
1	73.2	0.6	39.3	0.7	3565.6	41.4	1117.4	9.1	13.7	0.2	2.6	0.2	260.5	4.7	74.7	1.6	17.6	0.2	5.3	0.3
2	67.0	1.4	20.6	0.9	2409.6	31.1	940.6	18.4	8.5	0.1	2.7	0.2	211.7	4.4	135.7	3.5	13.9	0.2	5.8	0.3
3	64.3	1.1	30.9	1.0	3055.1	29.5	1767.4	26.7	12.2	0.2	6.3	0.4	299.4	4.6	283.4	7.7	18.1	0.2	11.0	0.5
4	52.1	0.3	14.7	0.6	2936.1	25.0	1364.2	22.8	21.8	0.3	16.7	1.7	736.0	12.9	510.2	11.5	32.3	0.3	23.6	1.2
5	57.0	1.1	13.6	0.6	2570.3	27.1	684.2	9.1	19.2	0.3	6.7	0.5	1225.8	23.6	967.5	20.1	24.8	0.3	13.2	0.7
6	59.5	0.6	15.1	0.6	2991.6	27.4	1682.6	27.8	7.1	0.3	5.1	0.5	289.6	2.2	161.7	5.5	9.9	0.3	8.8	1.3
7	33.9	0.7	14.2	0.5	2265.6	60.8	1058.6	15.0	15.7	0.3	9.2	0.7	426.0	7.6	349.2	11.3	19.9	0.2	14.2	0.9
8	56.2	0.9	36.0	2.3	2140.7	18.6	1197.5	10.3	10.2	0.2	2.6	0.2	325.6	2.4	89.9	1.8	19.0	0.3	3.1	0.3
9	142.7	3.5	56.4	1.6	2989.4	27.4	1966.8	19.8	23.4	0.7	6.1	0.4	282.6	5.6	107.3	2.2	25.6	0.7	7.5	0.4
10	322.3	6.9	90.5	3.7	5492.5	112.3	2622.5	113.9	43.1	1.0	6.2	0.3	844.1	16.8	354.7	15.1	26.0	0.5	4.6	0.3
11	233.8	5.8	54.8	1.8	4127.3	65.8	1645.4	50.5	37.1	1.2	7.4	0.4	595.6	11.3	203.5	2.4	24.5	1.1	5.6	0.3
12	184.9	4.8	56.1	1.9	3439.4	70.5	2213.5	57.7	16.3	0.4	4.7	0.4	260.7	5.3	150.5	3.5	11.3	0.2	5.7	0.3
13	160.5	4.4	71.4	2.0	2636.6	57.9	2471.5	49.8	9.4	0.2	5.5	0.4	307.7	3.2	149.1	3.4	8.7	0.2	6.0	0.4
14	228.3	4.6	23.3	0.7	4881.6	92.7	1361.5	46.2	38.5	0.7	5.5	0.4	1605.6	14.4	143.8	3.9	16.8	0.4	3.5	0.2
15	160.7	3.4	106.8	2.0	3740.4	57.9	2395.0	52.0	14.5	0.4	5.0	0.5	305.0	5.5	165.3	3.8	10.3	0.3	6.6	0.5
16	151.1	3.3	66.2	2.0	2636.4	63.3	1639.4	57.7	10.4	0.3	3.2	0.4	403.2	4.5	107.9	3.0	9.8	0.3	3.5	0.4

[a] Fossil = measurements of elemental signals corresponding with the body of the microfossil
[b] Background = the silica matrix of each sample, not associated with the body of the fossil
[c] Conc. = concentration in parts-per-million
[d] Err. = Error, which is the Poisson-per-pixel of the same area the concentration was measured from

End of Supplementary Material
